# Supplementary material for: Improving the quality of routine maternal and newborn data captured in primary health facilities in Gombe State, Northeastern Nigeria: a before-and-after study
Source: BMJ Open. 2020 Dec 2;10(12):e038174. doi: 10.1136/bmjopen-2020-038174 (PMC7713194; doi:10.1136/bmjopen-2020-038174)
Supplement: Supplementary data [file bmjopen-2020-038174supp001.pdf]

Supplementary data

Table S1. Data quality metrics and data sources reviewed

Data quality metric	Analysis/calculation	Source(s)
**Data quality dimension 1: Completeness and timeliness**		
Facility-level metrics		
Availability of monthly facility reports	Proportion of facility's expected monthly reports actually submitted	facility monthly reports (DHIS2)
Timeliness of monthly facility reports	Proportion of facility's expected monthly reports actually submitted on time	facility monthly reports (DHIS2)
Completeness of all 14 priority maternal and newborn health data elements, per monthly facility report	Proportion of facility's submitted reports that have a value reported for all 14 priority maternal and newborn health data elements	facility monthly reports (DHIS2)
Indicator-level metrics		
Completeness of data element	Proportion of non-missing values for a given data element in expected monthly reports	facility monthly reports (DHIS2)
**Data quality dimension 2: Internal consistency**		
Consistency over time	Ratio of value of indicator for the intervention period to the mean of preceding 3 years	facility monthly reports (DHIS2)
Outliers	Number and frequency of moderate outliers ($\pm$2-3SD from the mean) and extreme outliers ($\pm$3SD from the mean) of monthly values	facility monthly reports (DHIS2)
Consistency between related data elements	Reliability index (intraclass correlation coefficient, ICC) comparing two sets of related data elements	facility monthly reports (DHIS2)
Accuracy of facility reporting (data accuracy, concordance)	Reliability index (intraclass correlation coefficient, ICC) comparing original facility register count to monthly facility report in DHIS2	facility registers; facility monthly reports (DHIS2)
**Data quality dimension 3: External consistency**		
Consistency between household surveys and monthly facility reports	Ratio of indicator values in household surveys for facility catchment areas to matching facilities in DHIS2	household surveys; facility monthly reports (DHIS2)

Notes:
DHIS2=District Health Information Software version 2, SD=standard deviation, ICC=intraclass correlation coefficient.

S2. Consistency over time, 2015-2018 (Gombe State, n=492 facilities)

Indicator, data element		2015	2016	2017	2018	Mean (2015-2017)	Ratio of 2018 to Mean (2015-2017)	Mean (2015-2016)	Ratio of Mean (2017-2018) to Mean (2015-2016)
Main denominators									
First antenatal care visits		108,136	131,263	132,715	128,924	124,038	1.04	119,700	1.09
Total antenatal care visits		194,141	365,251	351,596	300,404	303,663	0.99	279,696	1.17
Facility deliveries		59,731	48,335	65,211	52,122	57,759	0.90	54,033	1.09
Data elements									
Antenatal care anemia testing	Number	47,809	58,514	68,189	97,128	58,171	**1.67**	53,162	**1.55**
	Coverage	44%	45%	51%	75%	47%	**1.61**	44%	**1.43**
Antenatal care syphilis testing	Number	26,231	24,569	34,461	47,393	28,420	**1.67**	25,400	**1.61**
	Coverage	24%	19%	26%	37%	23%	**1.60**	21%	**1.46**
Iron-folic acid supplementation	Number	172,447	317,420	248,404	282,221	246,090	1.15	244,934	1.08
	Coverage	89%	87%	71%	94%	82%	1.14	88%	0.94
At least one dose of intermittent preventive treatment of malaria	Number	32,960	27,209	30,292	76,482	30,154	**2.54**	30,085	**1.77**
	Coverage	30%	21%	23%	59%	25%	**2.40**	26%	**1.60**
At least one dose of tetanus toxoid vaccine	Number	69,374	76,754	83,058	85,293	76,395	1.12	73,064	1.15
	Coverage	64%	58%	63%	66%	62%	1.07	61%	1.05
Delivery by skilled birth attendant	Number	18,912	10,240	32,725	47,042	20,626	**2.28**	14,576	**2.74**
	Coverage	32%	21%	50%	90%	34%	**2.63**	26%	**2.66**
Live births and still births	Number	33,492	39,892	47,715	49,053	40,366	1.22	36,692	1.32
	Coverage	56%	83%	73%	94%	70%	1.35	69%	1.21
Baby weighed at birth	Number	32,719	39,145	46,850	48,162	39,571	1.22	35,932	1.32
	Coverage	55%	81%	72%	92%	69%	**1.34**	68%	**1.21**
Oral polio vaccine at birth	Number	50,587	60,636	66,895	64,457	59,373	1.09	55,612	1.18
	Coverage	85%	125%	103%	124%	104%	1.19	105%	1.08
Early postpartum-postnatal care within 3 days of birth	Number	3,930	6,265	8,575	12,868	6,257	**2.06**	5,098	**2.10**
	Coverage	7%	13%	13%	25%	11%	**2.27**	10%	**1.94**
Bacillus Calmette-Guérin vaccine given during postnatal period	Number	75,491	84,670	87,525	81,362	82,562	0.99	80,081	1.05
	Coverage	126%	175%	134%	156%	145%	1.07	151%	0.96

S3. Presence of outliers in facility summary reports in DHIS2 in Gombe State, July 2015-December 2018 (n=492 facilities)

	July 2015-March 2017		April 2017-Dec 2018	
**Indicator, data element**	**Moderate Outliers**	**Extreme Outliers**	**Moderate Outliers**	**Extreme Outliers**
First antenatal visits	1 (Jan 2017)	0	1 (May 2018)	0
Total antenatal visits	1 (Oct 2016)	0	1 (May 2018)	0
Facility deliveries	0	0	1 (May 2018)	0
Antenatal care anemia testing	0	0	1 (May 2018)	0
Antenatal care syphilis testing	0	0	0	0
Iron-folic acid supplementation	0	1 (Oct 2016)	1 (May 2018)	0
At least one dose of intermittent preventive treatment of malaria	0	1 (Oct 2016)	0	0
At least one dose of tetanus toxoid vaccine	1 (Oct 2016)	0	1 (May 2018)	0
Delivery by skilled birth attendant	1 (Mar 2017)	0	1 (May 2018)	0
Live births and still births	1 (Mar 2017)	0	1 (May 2018)	0
Baby weighed at birth	1 (Mar 2017)	0	1 (May 2018)	0
Oral polio vaccine at birth	0	0	1 (May 2018)	0
Early postpartum-postnatal care within 3 days of birth	1 (Jan 2017)	0	0	0
Bacillus Calmette-Guérin vaccine given during postnatal period	1 (Jan 2017)	0	1 (May 2018)	0
**TOTAL OUTLIERS**	**8**	**2**	**11**	**0**
	2 (Oct 2016), 3 (Jan 2017), 3 (Mar 2017)	2 (Oct 2016)	11 (May 2018)	0
