# Supplementary material for: Improving the quality of routine maternal and newborn data captured in primary health facilities in Gombe State, Northeastern Nigeria: a before-and-after study
Source: BMJ Open. 2020 Dec 2;10(12):e038174. doi: 10.1136/bmjopen-2020-038174 (PMC7713194; doi:10.1136/bmjopen-2020-038174)
Supplement: Author's manuscript [file bmjopen-2020-038174.draft_revisions.pdf]

BMJ Open

BMJ Open is committed to open peer review. As part of this commitment we make the peer review history of every article we publish publicly available.

When an article is published we post the peer reviewers' comments and the authors' responses online. We also post the versions of the paper that were used during peer review. These are the versions that the peer review comments apply to.

The versions of the paper that follow are the versions that were submitted during the peer review process. They are not the versions of record or the final published versions. They should not be cited or distributed as the published version of this manuscript.

BMJ Open is an open access journal and the full, final, typeset and author-corrected version of record of the manuscript is available on our site with no access controls, subscription charges or pay-per-view fees (http://bmjopen.bmj.com).

If you have any questions on BMJ Open's open peer review process please email info.bmjopen@bmj.com

BMJ Open

Improving the quality of routine data captured in primary health facilities through an integrated district-focused intervention in northeastern Nigeria

Journal:	*BMJ Open*
Manuscript ID	bmjopen-2020-038174
Article Type:	Original research
Date Submitted by the Author:	01-Mar-2020

Complete List of Authors:	Bhattacharya, Antoinette Alas; London School of Hygiene and Tropical Medicine Faculty of Infectious and Tropical Diseases, Department of Disease Control<br>Allen, Elizabeth; London School of Hygiene and Tropical Medicine Faculty of Epidemiology and Population Health, Department of Medical Statistics<br>Umar, Nasir; London School of Hygiene and Tropical Medicine Faculty of Infectious and Tropical Diseases, Disease Control<br>Audu, Ahmed; Gombe State Primary Health Care Development Agency<br>Felix, Habila; Gombe State Primary Health Care Development Agency<br>Schellenberg, Joanna; London School of Hygiene and Tropical Medicine, Disease Control<br>Marchant, Tanya; London School of Hygiene and Tropical Medicine Faculty of Infectious and Tropical Diseases, Disease Control

Keywords:	Health policy < HEALTH SERVICES ADMINISTRATION & MANAGEMENT, Maternal medicine < OBSTETRICS, PAEDIATRICS

SCHOLARONE™
Manuscripts

I, the Submitting Author has the right to grant and does grant on behalf of all authors of the Work (as defined in the below author licence), an exclusive licence and/or a non-exclusive licence for contributions from authors who are: i) UK Crown employees; ii) where BMJ has agreed a CC-BY licence shall apply, and/or iii) in accordance with the terms applicable for US Federal Government officers or employees acting as part of their official duties; on a worldwide, perpetual, irrevocable, royalty-free basis to BMJ Publishing Group Ltd ("BMJ") its licensees and where the relevant Journal is co-owned by BMJ to the co-owners of the Journal, to publish the Work in this journal and any other BMJ products and to exploit all rights, as set out in our licence.

The Submitting Author accepts and understands that any supply made under these terms is made by BMJ to the Submitting Author unless you are acting as an employee on behalf of your employer or a postgraduate student of an affiliated institution which is paying any applicable article publishing charge ("APC") for Open Access articles. Where the Submitting Author wishes to make the Work available on an Open Access basis (and intends to pay the relevant APC), the terms of reuse of such Open Access shall be governed by a Creative Commons licence – details of these licences and which Creative Commons licence will apply to this Work are set out in our licence referred to above.

Other than as permitted in any relevant BMJ Author's Self Archiving Policies, I confirm this Work has not been accepted for publication elsewhere, is not being considered for publication elsewhere and does not duplicate material already published. I confirm all authors consent to publication of this Work and authorise the granting of this licence.

For peer review only

Improving the quality of routine data captured in primary health facilities through an integrated district-focused intervention in northeastern Nigeria

Antoinette Alas Bhattacharya[1*], Elizabeth Allen[2], Nasir Umar[1], Ahmed Audu[3], Habila Felix[3], Joanna RM Schellenberg[1], and Tanya Marchant[1]

[1] Department of Disease Control, London School of Hygiene & Tropical Medicine, London, United Kingdom

[2] Department of Medical Statistics, London School of Hygiene & Tropical Medicine, London, United Kingdom

[3] State Primary Health Care Development Agency, Gombe, Nigeria

* Corresponding author

London School of Hygiene & Tropical Medicine

Keppel Street

London W1CE 7HT

United Kingdom

antoinette.bhattacharya@lshtm.ac.uk

+44 20 7636 8636

Word count: 3685

For peer review only - http://bmjopen.bmj.com/site/about/guidelines.xhtml

ABSTRACT

Introduction

Improving the quality of routine facility data is essential for local and national evidence-based monitoring of universal health coverage. We developed an integrated district-focused data quality intervention in a high mortality setting in northeastern Nigeria, and quantified change in the data quality metrics before and after the intervention.

Methods

Between April 2017-December 2018, we implemented an integrated data quality intervention in 11 local government areas (district-equivalent) overseeing 492 primary health facilities providing maternal and newborn care. We assessed 9 metrics across the data quality dimensions of completeness and timeliness, internal consistency, and external consistency. Data from facility registers, District Health Information Software version 2, and household surveys were used to assess these metrics for 14 maternal and newborn health data elements, comparing the 21-month period before the intervention (July 2015-March 2017) to the 21-month intervention period. We also considered whether assessing the data quality metrics beyond completeness and accuracy of facility reporting offered new insight into reviewing routine data quality.

Results

The data quality intervention was associated with improvements in 7 of 9 data quality metrics assessed including availability and timeliness of reporting, completeness of data elements, accuracy of facility reporting, consistency between related data elements, and frequency of outliers reported. Improvement differed by data element type, with content of care and commodity-related data improving more than contact-related data. Increases in the consistency between related data elements demonstrated improved internal consistency within and across facility documentation.

Conclusion

An integrated district-focused data quality intervention – including regular self-assessment of data quality, peer review and feedback, learning workshops, workplanning for improvement, and ongoing support through social media – can increase the completeness, accuracy, and internal consistency of facility-based routine data.

For peer review only - http://bmjopen.bmj.com/site/about/guidelines.xhtml

STRENGTHS AND LIMITATIONS OF THIS STUDY

- We extended the evidence on integrating data quality interventions within existing systems to improve the quality of facility-based data for monitoring and planning.
- We demonstrate the value of an integrated district-focused data quality intervention to include regular self-assessments of data quality, peer review and feedback, workplanning for improvement, and ongoing support through social media.
- We assessed the usefulness of the World Health Organization's catalogue of data quality metrics to measure and monitor the quality of routine facility data, as data quality studies primarily review completeness and accuracy of facility reporting.
- Without a concurrent comparison group, our before-and-after analyses cannot eliminate the effects of concurrent events and activities on data quality metrics.

For peer review only

INTRODUCTION

Routine health information systems provide essential data for governments and stakeholders to make decisions for managing performance and optimizing service delivery.[1-3] Routine health information systems, which include facility-based data, have the potential to provide disaggregated statistics important for understanding disparities and inequities in the provision of quality services and related health outcomes.[4 5]

Effective use of routine health data is dependent, in part, on the quality of data.[1 4 6-9] Studies assessing the quality of routine health data have shown persistent challenges in incomplete and untimely reporting, incomplete indicator-level data, inaccurate facility reporting, and imprecise target population estimates for coverage.[10-13] Further, studies have noted considerably poorer data

For peer review only - http://bmjopen.bmj.com/site/about/guidelines.xhtml

quality at the facility-level than at district-, state-, and national-levels, citing challenges in accurately capturing data as well as in tallying and summarizing service data for monthly reporting.[14-16]

Efforts to improve the quality of routine data have included trainings, workshops and review meetings, data verification surveys, strengthening feedback mechanisms, district- and case-based electronic information systems, and the provision of supplies and equipment such as facility registers and computers.[4 6 10 17-19] Activities which aligned with user priorities and were integrated within existing government systems were perceived to be advantageous as well as more likely to be adopted and adapted.[18]

Within the context of routine health information systems, the World Health Organization (WHO) has characterized routine data quality into four broad dimensions: completeness and timeliness; internal consistency; external consistency; and external comparisons.[20] While the abovementioned data quality interventions have demonstrated increases in completeness and accuracy of facility reporting[4 6 8 19 21], there are few peer-reviewed studies that quantitatively assessed changes in data quality metrics beyond this.[3 9 17]

In this study, our primary objective was to measure the changes in data quality metrics before and after the introduction of an integrated district-focused intervention in northeastern Nigeria for routine facility data captured in primary health facilities. A secondary objective was to determine the extent to which expanding data quality metrics beyond completeness and accuracy of facility reporting offered new insight into reviewing data quality.

METHODS

Study design

This was a before-and-after study design for a data quality intervention in all 11 local government areas (LGA, district-equivalent) of Gombe State, northeastern Nigeria. We present results for the

For peer review only - http://bmjopen.bmj.com/site/about/guidelines.xhtml

state (n=492 facilities), comparing the 21-month period before the intervention, July 2015-March 2017, with the 21-month period after introducing the intervention, April 2017-December 2018.

Study approvals

Study approvals were obtained from the London School of Hygiene & Tropical Medicine (reference 14091) and the Health Research Ethics Committees for Nigeria (reference NHREC/01/01/2007) and Gombe State (reference ADM/S/658/Vol. II/66).

Patient and public involvement

Patients or the public were not involved in the design, conduct, reporting, or dissemination of the research described here.

Study setting

Gombe State is located in northeastern Nigeria, a region with high maternal and newborn mortality at 1,549 per 100 000 live births and 35 per 1,000 live births, respectively.[22 23] With an estimated population of 2.9 million, Gombe is predominantly rural and 35%% of the women have some primary school education.[24 25] Most women access maternity care through public facilities. Seventy-two percent of women reported at least one antenatal care visit during their last pregnancy and 28% gave birth in a health facility.[23 25] In 2018, over 70% of facility deliveries took place in rural primary health facilities.[26]

Under Nigeria's national policy of *Primary Health Care Under One Roof*, the Gombe State Primary Health Care Development Agency oversees the administration and service delivery for primary health facilities across 11 local government areas (LGA); each LGA has 10-11 political wards (114

For peer review only - http://bmjopen.bmj.com/site/about/guidelines.xhtml

wards, total).[27 28] LGA monitoring and evaluation officers are responsible for community- and facility-level data collection, validation, and reporting to the state office. LGA maternal, neonatal, and child health (MNCH) coordinators support the supervision and implementation of services for women and children.

During the intervention period, Gombe State had 492 primary health facilities providing antenatal and childbirth services. As in other states in Nigeria, Gombe facility staff generally completed 13 paper-based registers to document the services they provide (Nigeria Health Management Information Systems, version 2013). Every month, a subset of data in these registers were tallied and summarized in a paper-based report and sent to the LGA health office to be entered into District Health Information Software, version 2 (DHIS2).

Data quality intervention

The routine data quality intervention period spanned 21 months, from April 2017 through December 2018. The intervention emphasized the partnership between the LGA monitoring and evaluation officer and the LGA maternal, newborn, and child health program coordinator to underscore the link between the quality and use of routine data.[8] It was designed as a facilitative layer to existing LGA-level supervision responsibilities, leveraging scheduling of ongoing activities to minimize cost, adding job aids and defining performance standards to provide structure to data quality checking duties and to target feedback to facilities.

The intervention included the following activities: (i) data quality learning workshops to present data quality self-assessment findings and develop workplans for improvement; (ii) defining data quality performance standards and milestones for completeness, timeliness, and consistency; (iii) introduction of job aids to self-assess data quality according to the WHO data quality metrics; (iv) monthly state- and LGA-level data quality summary reports; (v) intentional practice on providing

For peer review only - http://bmjopen.bmj.com/site/about/guidelines.xhtml

constructive feedback to peers and low-performing facilities to promote a positive culture of data use; and (vi) ongoing engagement on data quality issues through government-approved communication channels, including the social media application WhatsApp.

Twenty-six main attendees participated in the workshops and ongoing communication in-between workshops. This included two participants from each of the 11 LGAs, a monitoring and evaluation officer and the maternal, newborn, and child health program coordinator. At the state-level, four officials participated: the director of the state planning, research, and statistics department, the state monitoring and evaluation officer, the state health management information systems officer, and the state maternal, newborn, and child health coordinator.

Four data quality learning workshops took place every six to nine months. The two-day workshops included the introduction of job aids and practical sessions to strengthen data quality checking skills, the presentation of the state's and each LGA's self-assessment of data quality, and the development of six-month workplans to improve the quality of facility-based routine data. Using materials designed for post-graduate learning and teaching, there was intentional practice on how to provide constructive feedback to peers and facilities to promote a positive culture of information use.[29]

At each workshop, a major theme emerged during the workplanning sessions (Figure 1). For example, at the first workshop, participants were concerned with inconsistencies observed between the paper-based facility registers and the facility's monthly summary reports. Activities enacted from the workplanning session were to revitalize dormant groups previously set up to address program monitoring and evaluation activities: (i) LGA data validation committee meetings, where facilities bring their registers for verification against their submitted monthly facility report. and (ii) a social media WhatsApp group of LGA actors and facilities. The LGA teams posted pictures and comments on these facility interactions on the WhatsApp group for encouragement and accountability.

After the first workshop, the Gombe State monitoring and evaluation officer disseminated monthly state- and LGA-level data quality summary reports. LGAs were assessed according to the WHO data

For peer review only - http://bmjopen.bmj.com/site/about/guidelines.xhtml

quality metrics and recommendations for improvement were offered. Initially, this activity was designed for the external workshop facilitators to compose and disseminate, while building the capacity of the state officer to take on this task over time.

Outcomes

Using the WHO data quality review toolkit for routine facility data, we assessed 9 metrics across the three data quality dimensions of completeness and timeliness; internal consistency; and external consistency.[30] The data sources and analyses for each data quality metric are described in the following section. Supplementary table S1 provides additional information on each data quality metric assessed and the data sources reviewed.

Data analysis and data sources

Three data sources were used to assess the routine data quality metrics, described below: facility-reported data in DHIS2; external facility surveys; and external household surveys.

DHIS2 contained monthly reports for the 492 primary facilities providing antenatal care, childbirth, and postnatal care services. Monthly aggregated DHIS2 data for July 2015-December 2018 were downloaded at one time and included 14 maternal and newborn health-related data elements. These data were used to assess availability of facility reporting; timeliness of facility reporting; completeness of all 14 priority maternal and newborn health data elements, per monthly facility report; completeness of data element; presence of moderate and extreme outliers; consistency of indicator values over time; and consistency between related data elements. We calculated the intraclass correlation coefficient (ICC) as a measure of reliability for the consistency between related data elements. ICC values range between 0 and 1, where values approaching 1 represented greater reliability.

For peer review only - http://bmjopen.bmj.com/site/about/guidelines.xhtml

In 2016 and 2018, facility-level surveys were conducted in 97 primary facilities across Gombe to assess their readiness to provide maternal and newborn health services. The two surveys represented the approximate midpoints of the pre-intervention and intervention period. The selected facilities were a state-wide random sample drawn from all primary health facilities. Detailed methods are reported elsewhere.[31] The facility survey protocol was similar to a Service Availability and Readiness Assessment and also included data extraction from the facility's paper-based antenatal and postnatal care register and the labor and delivery register (Nigeria health management information system, version 2013).[32] A trained third party data collection team tallied and recorded the register data for each month of the six-month periods immediately prior to the survey: January-June 2016 and February-July 2018.

These data were used to assess the accuracy of facility reporting (also referred to as data accuracy, data verification, or concordance in peer-reviewed literature). We compared the facilities' paper-based registers data with the facilities' monthly reported data in DHIS2. As with the consistency between related data elements, we calculated the ICC as a measure of reliability for the facility's reporting of the indicator in DHIS2. ICC values approaching 1 represented greater reliability.

In 2016 and 2018, household-level surveys were conducted in the catchment areas of the abovementioned 97 primary facilities to assess access to and quality of maternal and newborn services.[31] These catchment areas represented 79 enumeration areas since some enumeration areas were served by more than one facility. All households in each enumeration area were surveyed. The household surveys included a mother's module which asked all women who reported a birth in the last year a detailed set of questions about their contact with health services across the continuum of care from pregnancy to postnatal care. Informed consent was obtained at the community leadership-level and at the individual-level for each respondent. All invited participants agreed be interviewed.

These data were used for external consistency during the pre-intervention and intervention periods. We compared coverage estimates from household surveys to those from the 97 matching facilities in

For peer review only - http://bmjopen.bmj.com/site/about/guidelines.xhtml

DHIS2. We compared the same recall period for the surveys and the DHIS2. The DHIS2 data are considered consistent if they fall within the confidence intervals of the external household survey estimates.

Calculations of point estimates and their 95% confidence intervals were done using the svyset Stata command (StataCorp, College Station, USA) to adjust for clustering. We chose the highest-order clustering level to provide the most conservative confidence interval estimates.[33]

For peer review only

RESULTS

An integrated district-focused data quality intervention was implemented across 11 LGAs overseeing 492 primary health facilities providing maternal and newborn care services. Below, we present the results for 9 data quality metrics.

Completeness and timeliness

Table 1 summarizes the completeness and timeliness of reporting at the facility- and indicator-levels.

At the facility-level, the availability of monthly facility reports improved from 72% to 82% (p<0.001) and timeliness of submitting the reports increased from 60% to 72% (p<0.001). The proportion facility-months where all 14 priority maternal and newborn health data elements contained a value within the monthly report increased from 62% to 68% (p<0.001).

At the indicator-level, seven of 14 data elements assessed improved in completeness compared to the pre-intervention period. Indicator-level completeness did not change for contact indicators such as first antenatal care visits, total antenatal care visits, and facility deliveries.

For peer review only - http://bmjopen.bmj.com/site/about/guidelines.xhtml

Table 1. Facility- and indicator-level completeness and timeliness, Gombe State (n=492 facilities)

	Pre-intervention Jul '15-Mar '17 % (95% CI)	Intervention Apr '17- Dec '18 % (95% CI)
**Facility-level**		
Availability of monthly facility reports	72 (69-74)	82 (80-84)
Timeliness of monthly facility reports	60 (57-62)	72 (70-74)
Completeness of all 14 priority maternal and newborn health data elements, per monthly facility report	62 (60-63)	68 (66-70)
**Indicator-level**		
For every 100 facilities that submitted a monthly facility report, the percentage of facilities reporting a value for the following services:		
First antenatal care visits	76 (67-85)	77 (70-84)
Total antenatal care visits	100 (99-100)	100 (100-100)
Facility deliveries	68 (59-77)	67 (60-74)
For every 100 facilities that reported a value for first ANC visit, the percentage of facilities reporting a value for the following services:		
Antenatal care anemia testing	28 (16-39)	36 (24-49)
Antenatal care syphilis testing	42 (23-61)	29 (23-35)*
Iron-folic acid supplementation	80 (75-84)	89 (85-92)
At least one dose administered of intermittent preventive treatment of malaria	45 (34-56)	56 (49-62)
At least one dose administered of tetanus toxoid vaccine	90 (86-93)	89 (86-91)
For every 100 facilities that reported a value for a facility delivery, the percentage of facilities reporting a value for the following services:		
Delivery by skilled birth attendant	43 (25-61)	86 (81-91)
Live birth or still birth	90 (86-95)	96 (94-97)
Baby weighed at birth	89 (83-95)	95 (94-97)
Oral polio vaccine given at birth	79 (70-87)	86 (82-90)
Early postpartum-postnatal care within 3 days of birth	45 (38-53)	55 (46-64)
Bacillus Calmette-Guérin vaccine given during postnatal care period	79 (71-88)	81 (77-86)

Notes:
* During the intervention period, commodities for antenatal care syphilis testing were redistributed and restricted to 57 facilities. For these 57 facilities, completeness of data for antenatal care syphilis testing increased from 48% (95% CI: 28-68) to 77% (95% CI: 69-86).

For peer review only - http://bmjopen.bmj.com/site/about/guidelines.xhtml

Internal consistency: consistency between related data elements

To assess the consistency between related data elements with a predictable relationship, two types of relationships were reviewed (Figure 2). The first type of relationship assessed concurrent tallying across different data elements within and across facility registers. For example, (1) normal deliveries + caesarean deliveries + assisted deliveries = live births + still births; and (2) total postpartum visits reported = sum of the postnatal visit categories reported. For Gombe State, the ICC of delivery types (normal, caesarean, assisted) to birth types (live births, still births) improved from 0.78 (95%CI: 0.67-0.85) to 0.95 (0.91-0.97). Similar patterns of improvement were noted for postnatal visit tallying from an ICC of 0.54 (0.38-0.65) to 0.87 (0.74-0.93).

The second type of relationship assessed was a service provision compared to a contact indicator (e.g., the number of antenatal care syphilis testing done compared to antenatal care first visits, the number of babies weighed at birth compared to the number of facility deliveries). During the pre-intervention period, one of the 10 relationships reflected high consistency: iron-folic acid supplementation. During the implementation period, five of the 10 relationships reflected improved consistency.

Internal consistency: accuracy of facility reporting

Comparing the facilities' registers with their submitted monthly reports, accuracy of facility reporting (data accuracy) had improved for 6 of 7 indicators, reflecting greater agreement during the intervention period (Figure 3). For total postnatal care visits, considerable variation between facilities can be seen with an ICC of 0.59 (95% CI: 0.44-0.71) during the intervention period.

Internal consistency: outliers and consistency over time

For peer review only - http://bmjopen.bmj.com/site/about/guidelines.xhtml

Supplementary tables S2 and S3 summarize the presence of outliers and the consistency over time for the 14 maternal and newborn data elements. The frequency of months when outliers were reported decreased during the intervention period. However, 11 moderate outliers were reported during the intervention period compared to eight moderate outliers and two extreme outliers reported during the pre-intervention period. All 11 outliers reported during the intervention period occurred in May 2018 during a health worker strike. Of the 14 data elements assessed, six data elements were inconsistent over time due to reported increases in services when comparing the final year of the intervention 2018 to the mean value of the last 3 years and when comparing the pre-intervention and intervention periods.

External consistency: agreement between facility summary reports in DHIS2 and household surveys

Figure 4 summarizes external consistency, which is the agreement between facility-based routine data in DHIS2 compared to household-level surveys in the catchment areas of these facilities. Other than the indicator for early postpartum-postnatal care, there was no agreement nor any consistent pattern of agreement between facility-based routine data and the household surveys. DHIS2 underestimated compared to the household survey for at least one dose of intermittent preventative therapy for malaria in pregnancy and at least one dose of tetanus toxoid. DHIS2 overestimated compared to the household survey for baby weighed at birth, oral polio vaccine given at birth, and Bacillus Calmette-Guérin given during postnatal period. For antenatal care anemia testing, facility-based estimates were within the household survey estimate confidence interval but overestimated compared to the household survey during the intervention period.

For peer review only - http://bmjopen.bmj.com/site/about/guidelines.xhtml

DISCUSSION

Facility-based routine data are an important source for monitoring, performance management, and planning.[9] Our study found that an integrated district-focused data quality intervention – which included regular self-assessment of data quality, peer review and feedback, learning workshops, workplanning for improvement, monthly data quality reports, and ongoing support through social media – was associated with improvements across most WHO data quality metrics. There were differences in data quality improvement by data element type.[34] Data related to content of care or the provision of commodities, such as syphilis testing and intermittent preventative therapy for malaria, improved more across data quality metrics compared to contact indicators which had relatively higher data quality metrics before the intervention, such as first antenatal care visits and facility deliveries.

This was an integrated data quality intervention designed to facilitate existing state- and district-level data quality checking responsibilities and emphasize the partnership between the monitoring and evaluation officers and the maternal, newborn and child health program coordinators to expand local access to the DHIS2 data, use the data, and problem solve.[9] The evolution of the intervention through the workplanning sessions prompted local solutions defined by the participants as feasible and within their resources to implement. In particular, the participants' decision to revitalize the data validation committees during the first workshop engaged the facilities early on to ensure the facility's register counts matched the facility's monthly report aggregate value. This early engagement with facilities could have contributed to the increased ICCs for accuracy of facility reporting observed during the intervention period (as shown in Figure 3). A formative phase of the intervention might have captured activities such as the data validation committee as a pre-defined intervention activity. Including a formative phase should be a consideration for future implementation.

For peer review only - http://bmjopen.bmj.com/site/about/guidelines.xhtml

Our findings aligned with previous studies reporting improvements in completeness, timeliness, and accuracy of facility reporting after intervention. However, this study's relative gains may reflect the scale of working through 11 districts with 492 primary health facilities.[4 6 8 19 21] A data quality intervention in KwaZulu-Natal province, South Africa, which included trainings, monthly data meetings, and external data quality audits across 78 facilities improved completeness of six data elements from 26% to 64% and the agreement between facility records and reports (data accuracy) from 37% to 65%.[6] A province-wide data quality intervention in Sofala, Mozambique, for 26 facilities included regular district-level review meetings for health workers and managers, data dashboards for tracking trends and rankings, human resource optimization models, and equipment purchase and maintenance. The summary measure used to evaluate data quality improvement, concordance, improved from 56% to 88% during the intervention period.[4] The introduction of an electronic medical record to support data quality improvement in 27 facilities across Kenya recorded a decline in missing data from 31% to 13% for 24 data elements, with a mean concordance score increasing across facilities by 1.79 (95% CI:0.25-3.33).[19]

While our findings align with previous studies for increased completeness, timeliness, and accuracy, our study reviewed additional WHO metrics to give a more comprehensive picture of the dimensions of data quality. This study also provided an opportunity to reflect on the relative usefulness of assessing all WHO data quality metrics to understand the quality of routine data in a given context. Sharp increases in service uptake due to health campaigns or targeted health projects make the assessment of moderate outliers and consistency over time less insightful about data quality, especially in the context of urgent efforts towards achieving universal health coverage. Our study found that, other than early postpartum-postnatal care within three days of birth, there were no instances of agreement between the facility-based routine data and external household surveys. However, an emerging body of criterion validity studies have demonstrated mixed results in the ability of women to recall facility-based pregnancy- and childbirth-related events in household

For peer review only - http://bmjopen.bmj.com/site/about/guidelines.xhtml

For peer review only

surveys.[35-39] More research is needed on how to reconcile health facility and household survey data, while also reconsidering the emphasis on the household surveys as the reference standard.

The data quality metric to assess the consistency between related data elements provided useful insights in addition to the completeness and accuracy regularly reported in the literature. Assessing the data relationship for a service provision compared to a contact indicator (e.g., ANC anemia testing compared to ANC first visits) allows for discussion on whether observed discrepancies are due to low service uptake or poor reporting, an important consideration given the emphasis on improving quality of care and understanding effective coverage.[40] Further, assessing data relationships that require concurrent tallying of services/information across data sources (e.g. facility attendance = inpatient + outpatient; normal delivery + caesarean delivery + assisted delivery = live births + still births), provides useful insight about whether a facility is paying attention to the internal consistency of their data within and across facility documentation. Focus on accuracy of facility reporting, a more common metric assessed in the peer-reviewed literature (referred to as data accuracy or concordance), is an important data quality metric as subnational-, national-, and global-level monitoring cannot take place effectively without the confidence that the facilities have summarized and tallied the data as intended. However, this focus on the accuracy of facility reporting up through the different levels of the health system do not require that these data be internally consistent with other data.

Our study had limitations. Without a concurrent comparison group, our before-and-after analyses cannot eliminate the effects of concurrent events and activities on data quality metrics. It is possible that other activities contributed to the observed data quality improvements. Given the high burden of maternal and neonatal mortality, the Gombe State Primary Health Care Development Agency spearheaded an initiative to improve maternal and neonatal services with the aim of having one fully functional primary health facility in each of its 114 wards. During the intervention period, 57 facilities (12% of the 492 maternity facilities) received support including facility-level quality improvement

For peer review only - http://bmjopen.bmj.com/site/about/guidelines.xhtml

support as well as community-based outreach and education to increase uptake of services. Facility-level activities included support on data quality to monitor trends in services provided and the provision of computers and facility registers. Additionally, similar to other data quality assessments, we did not validate the data through direct clinical observations[4 6 10 12 41-43] nor did we compare the paper-based monthly summary reports to their electronic versions in DHIS2[6 12 17 44-46]. Despite close attention to quality control, the facility- and household-level surveys might still be susceptible to errors in data recording, including incorrectly tallying the number of events in the original facility registers for comparison with data in DHIS2.

Improving the quality of routine facility data is essential for local and national evidence-based monitoring of universal health coverage. We found that an integrated district-focused data quality intervention was associated with increases across most WHO data quality metrics for routine facility-based data. Future initiatives should aim to incorporate national- and higher subnational-levels of the health system to determine scalability and sustainability of integrated data quality interventions in the long-term.

Acknowledgement

The authors wish to acknowledge the leadership of the Gombe State Primary Health Care Development Agency throughout the development and implementation of the data quality intervention. We also appreciate the cooperation of and partnership with the LGA M&E officers, MNCH coordinators, and Gombe State implementing partners to improve the quality of routine maternal and newborn health data in Gombe State. We are grateful to the women who participated in the household surveys and to the team who conducted the facility and household data collection. Finally, we are grateful to the facility teams responsible for providing and documenting their care for women and newborns.

For peer review only - http://bmjopen.bmj.com/site/about/guidelines.xhtml

Contributors

AAB and TM conceived and designed the study. AAB and EA designed the analyses. AAB carried out the analyses and composed the initial draft. JS, TM, EA, NU, AA, HF reviewed the early drafts. All authors approved the final draft.

Data sharing statement

Data for this study are available from the London School of Hygiene & Tropical Medicine public repository: http://datacompass.lshtm.ac.uk/229/.

Funding

This work was supported by the Bill & Melinda Gates Foundation [OPP1149259]. The funder of this study had no role in the study's design or conduct, data collection, analysis or interpretation of results, writing of the paper, or decision to submit for publication.

Competing interests

Mr Felix Habila and Mr Ahmed Audu are members of the Gombe State Primary Health Care Development Agency. The authors declare no other competing interests.

For peer review only

For peer review only - http://bmjopen.bmj.com/site/about/guidelines.xhtml

References

1. Nutley T. Improving data use in decision making: an intervention to strengthen health systems. Chapel Hill, North Carolina: MEASURE Evaluation, 2012.
2. AbouZahr C, Boerma T. Health information systems: the foundations of public health. *Bull World Health Organ* 2005;83(8):578-83.
3. Nisingizwe MP, Iyer HS, Gashayija M, et al. Toward utilization of data for program management and evaluation: quality assessment of five years of health management information system data in Rwanda. *Global Health Action* 2014;7:25829. doi: http://dx.doi.org/10.3402/gha.v7.25829
4. Wagenaar BH, Gimbel S, Hoek R, et al. Effects of a health information system data quality intervention on concordance in Mozambique: time-series analyses from 2009–2012. *Population Health Metrics* 2015;13(1):9. doi: 10.1186/s12963-015-0043-3
5. Chilundo B, Sundby J, Aanestad M. Analysing the quality of routine malaria data in Mozambique. *Malar J* 2004;3(3) doi: 10.1186/1475-2875-3-3
6. Mphatswe W, Mate KS, Bennett B, et al. Improving public health information: a data quality intervention in KwaZulu-Natal, South Africa. *Bull World Health Organ* 2012;90(3):176-82. doi: https://dx.doi.org/10.2471/BLT.11.092759
7. Mutale W, Chintu N, Amoroso C, et al. Improving health information systems for decision making across five sub-Saharan African countries: implementation strategies from the African Health Initiative. *BMC Health Serv Res* 2013;13(2):S9. doi: 10.1186/1472-6963-13-S2-S9
8. Braa J, Heywood A, Sahay S. Improving quality and use of data through data-use workshops: Zanzibar, United Republic of Tanzania. *Bull World Health Organ* 2012;90(5):379-84. doi: https://dx.doi.org/10.2471/BLT.11.099580
9. Maïga A, Jiwani SS, Mutua MK, et al. Generating statistics from health facility data: the state of routine health information systems in Eastern and Southern Africa. *BMJ Global Health* 2019;4(5):e001849. doi: 10.1136/bmjgh-2019-001849
10. Bosch-Capblanch X, Ronveaux O, Doyle V, et al. Accuracy and quality of immunization information systems in forty-one low income countries. *Trop Med Int Health* 2009;14(1):2-10. doi: 10.1111/j.1365-3156.2008.02181.x
11. Ronveaux O, Rickert D, Hadler S, et al. The immunization data quality audit: verifying the quality and consistency of immunization monitoring systems. *Bull World Health Organ* 2005;83(7):503-10.
12. Mate KS, Bennett B, Mphatswe W, et al. Challenges for routine health system data management in a large public program to prevent mother-to-child HIV transmission in South Africa. *PLoS One* 2009;4(5):e5483.
13. Maina I, Wanjala P, Soti D, et al. Using health-facility data to assess subnational coverage of maternal and child health indicators, Kenya. *Bull World Health Organ* 2017;95(10):683-94. doi: 10.2471/BLT.17.194399
14. Belay H, Lippeveld T. Inventory of PRISM framework and tools: application of PRISM tools and interventions for strengthening routine health information system performance. Working Paper Series No: WP-13-138. Chapel Hill, North Carolina, USA: MEASURE Evaluation, 2013.
15. Hotchkiss DR, Aqil A, Lippeveld T, et al. Evaluation of the Performance of Routine Information System Management (PRISM) framework: evidence from Uganda. *BMC Health Serv Res* 2010;10:188.
16. Aqil A, Avila JE, Mejia LSP, et al. Guanajuato SINAIS assessment. Technical Report No: SR-10-60. Chapel Hill, North Carolina, USA: MEASURE Evaluation, 2010.
17. Gimbel S, Micek M, Lambdin B, et al. An assessment of routine primary care health information system data quality in Sofala Province, Mozambique. *Population Health Metrics* 2011;9:12.
18. Gimbel S, Mwanza M, Nisingizwe MP, et al. Improving data quality across 3 sub-Saharan African countries using the Consolidated Framework for Implementation Research: results from the

For peer review only - http://bmjopen.bmj.com/site/about/guidelines.xhtml

African Health Initiative. *BMC Health Serv Res* 2017;17(Suppl 3):828. doi: 10.1186/s12913-017-2660-y
19. Muthee V, Bochner AF, Osterman A, et al. The impact of routine data quality assessments on electronic medical record data quality in Kenya. *PLoS One* 2018;13(4):e0195362. doi: https://dx.doi.org/10.1371/journal.pone.0195362
20. MEASURE Evaluation. Routine health information systems: a curriculum on basic concepts and practices. Chapel Hill, North Carolina, USA: MEASURE Evaluation, 2017.
21. Gething P, Noor A, Gikandi P, et al. Improving imperfect data from health management information systems in Africa using space-time geostatistics. *PLoS Med* 2006;3 doi: 10.1371/journal.pmed.0030271
22. Izugbara CO, Wekesah FM, Adedini SA. Maternal health in Nigeria: a situation update. Nairobi, Kenya: African Population and Health Research Center, 2016.
23. National Bureau of Statistics and United Nations Children's Fund. Multiple Indicator Cluster Survey 2016-17, Survey Findings Report. Abuja, Nigeria: National Bureau of Statistics and United Nations Children's Fund, 2017.
24. Gombe State Government. History of Gombe State, Nigeria 2018 [Available from: http://gombestate.gov.ng/history-2/ accessed September 9, 2019.
25. National Population Commission NPC, Icf. Nigeria Demographic and Health Survey 2018 - Final Report. Abuja, Nigeria: NPC and ICF, 2019.
26. Nigeria District Health Information System version 2. Nigeria Health Management Information System, version 2013: Gombe State facility deliveries, by facility type, January-December 2018. Abuja: Nigeria District Health Information System, version 2, 2019.
27. Federal Government of Nigeria. Integrating primary healthcare governance in Nigeria: implementation manual, 2013.
28. National Primary Health Care Development Agency. Integrating primary health care governance in Nigeria (PHC Under One Roof). Abuja: NPHCDA, 2013.
29. Lippeveld T. Routine health facility and community information systems: creating an information use culture. *Global Health: Science and Practice* 2017;5(3):338–40.
30. World Health Organization. Data quality review: a toolkit for facility data quality assessment. Geneva: World Health Organization, 2017.
31. Marchant T. Change in maternal and newborn health care. Interactions between families and frontline workers – their frequency, quality, and equity – and coverage of interventions for mothers and newborns. Report from Gombe State, Nigeria, 2012-2015. IDEAS Project Report: London School of Hygiene and Tropical Medicine, 2016.
32. World Health Organization. Service Availability and Readiness Assessment (SARA): an annual monitoring system for service delivery. Reference Manual, Version 2.2. Geneva: World Health Organization, 2015.
33. Bottomley C, Kirby MJ, Lindsay SW, et al. Can the buck always be passed to the highest level of clustering? *BMC Med Res Methodol* 2016;16:29-29. doi: 10.1186/s12874-016-0127-1
34. Bhattacharya AA, Umar N, Audu A, et al. Quality of routine facility data for monitoring priority maternal and newborn indicators in DHIS2: A case study from Gombe State, Nigeria. *PLoS One* 2019;14(1):e0211265. doi: https://dx.doi.org/10.1371/journal.pone.0211265
35. Stanton CK, Rawlins B, Drake M, et al. Measuring coverage in MNCH: Testing the validity of women's self-report of key maternal and newborn health interventions during the peripartum period in Mozambique. *PLoS One* 2013;8(5):e60694. doi: 10.1371/journal.pone.0060694
36. Blanc AK, Warren C, McCarthy KJ, et al. Assessing the validity of indicators of the quality of maternal and newborn health care in Kenya. *Journal of Global Health* 2016;6(1):010405. doi: 10.7189/jogh.06.010405

For peer review only - http://bmjopen.bmj.com/site/about/guidelines.xhtml

37. Blanc AK, Diaz C, McCarthy KJ, et al. Measuring progress in maternal and newborn health care in Mexico: validating indicators of health system contact and quality of care. *BMC Pregnancy Childbirth* 2016;16(1):255. doi: 10.1186/s12884-016-1047-0
38. McCarthy KJ, Blanc AK, Warren CE, et al. Women's recall of maternal and newborn interventions received in the postnatal period: a validity study in Kenya and Swaziland. *Journal of Global Health* 2018;8(1):010605-05. doi: 10.7189/jogh.08.010605 [published Online First: 2018/05/12]
39. Bhattacharya AA, Allen E, Umar N, et al. Monitoring childbirth care in primary health facilities: a validity study in Gombe State, northeastern Nigeria. *Journal of Global Health* 2019;9(2):020411-11. doi: 10.7189/jogh.09.020411
40. Amouzou A, Leslie HH, Ram M, et al. Advances in the measurement of coverage for RMNCH and nutrition: from contact to effective coverage. *BMJ Global Health* 2019;4(Suppl 4):e001297. doi: 10.1136/bmjgh-2018-001297
41. Puttkammer N, Baseman JG, Devine EB, et al. An assessment of data quality in a multi-site electronic medical record system in Haiti. *Int J Med Inform* 2016;86:104-16.
42. Glele Ahanhanzo Y, Ouendo E-M, Kpozehouen A, et al. Data quality assessment in the routine health information system: an application of the lot quality assurance sampling in Benin. *Health Policy Plan* 2015;30(7):837-43.
43. Makombe SD, Hochgesang M, Jahn A, et al. Assessing the quality of data aggregated by antiretroviral treatment clinics in Malawi. *Bull World Health Organ* 2008;86(4):310-4.
44. Nicol E, Dudley L, Bradshaw D. Assessing the quality of routine data for the prevention of mother-to-child transmission of HIV: an analytical observational study in two health districts with high HIV prevalence in South Africa. *Int J Med Inform* 2016;95:60-70.
45. Mlambo MG, Peltzer K, Koivu A. Prevention of mother-to-child transmission of HIV data completeness and accuracy assessment in health facilities of the Nkangala District. *Health SA Gesondheid* 2014;19(1):a774.
46. Omoleke SA, Tadesse MG. A pilot study of routine immunization data quality in Bunza Local Government area: causes and possible remedies. *The Pan African Medical Journal* 2017;27:239. doi: https://dx.doi.org/10.11604/pamj.2017.27.239.11875

For peer review only

For peer review only - http://bmjopen.bmj.com/site/about/guidelines.xhtml

Figures

Figure 1. Data quality learning workshops in Gombe State, April 2017-December 2018

Figure 2. Internal consistency: consistency between data elements with a predictable relationship (n=492 facilities)
Notes: ICC values range from 0 to 1, with values approaching 1 representing greater reliability.

Figure 3. Accuracy of facility reporting: comparison of paper-based facility records and facility monthly reports in DHIS2 (n=97 facilities)
Notes: ICC values range from 0 to 1, with values approaching 1 representing greater reliability.

Figure 4. External consistency: comparison of household-level survey and facility summary reports in DHIS2 (n=97 facilities)

For peer review only

For peer review only - http://bmjopen.bmj.com/site/about/guidelines.xhtml

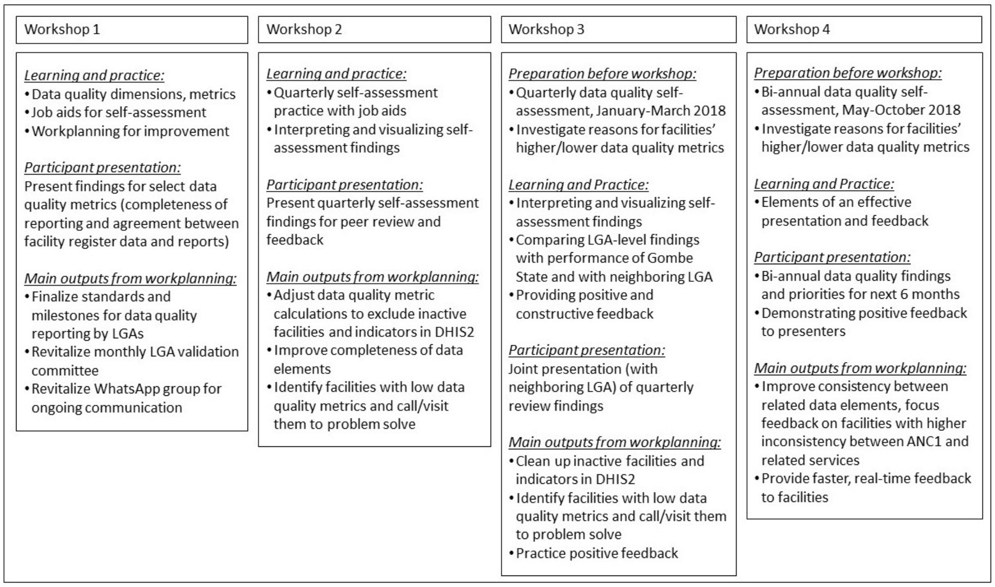

Figure 1. Data quality learning workshops in Gombe State, April 2017-December 2018

322x189mm (96 x 96 DPI)

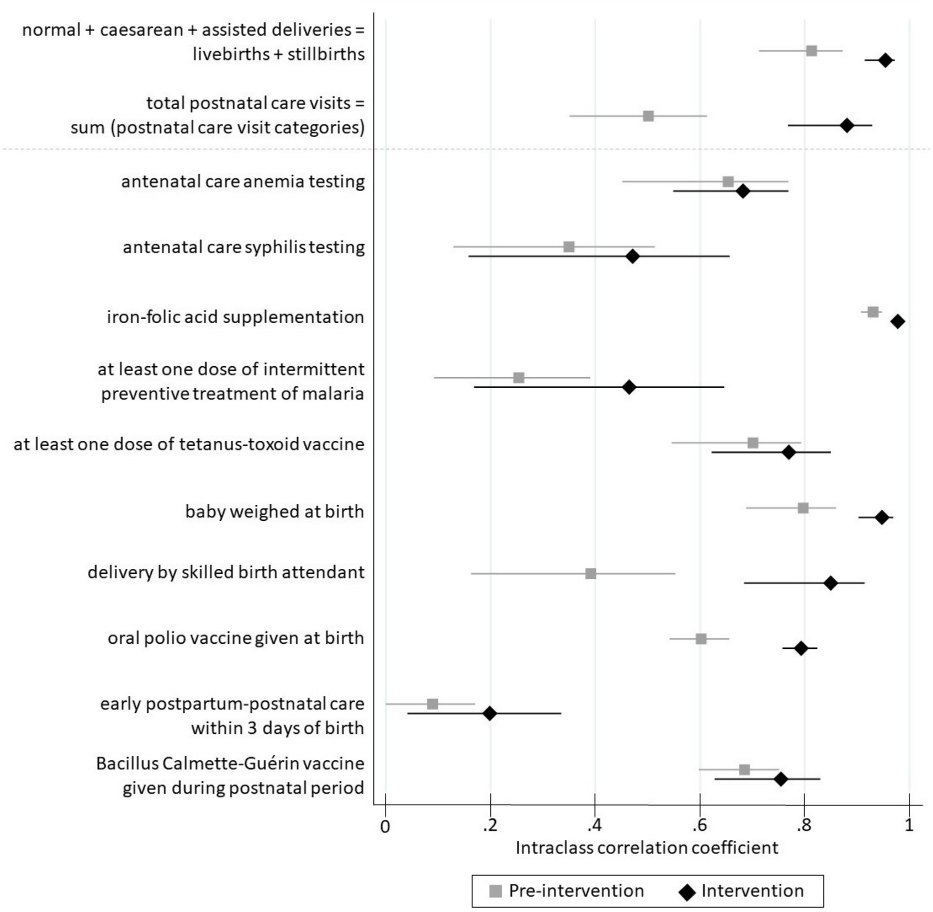

Figure 2. Internal consistency: consistency between data elements with a predictable relationship (n=492 facilities)
Notes: ICC values range from 0 to 1, with values approaching 1 representing greater reliability.

314x300mm (96 x 96 DPI)

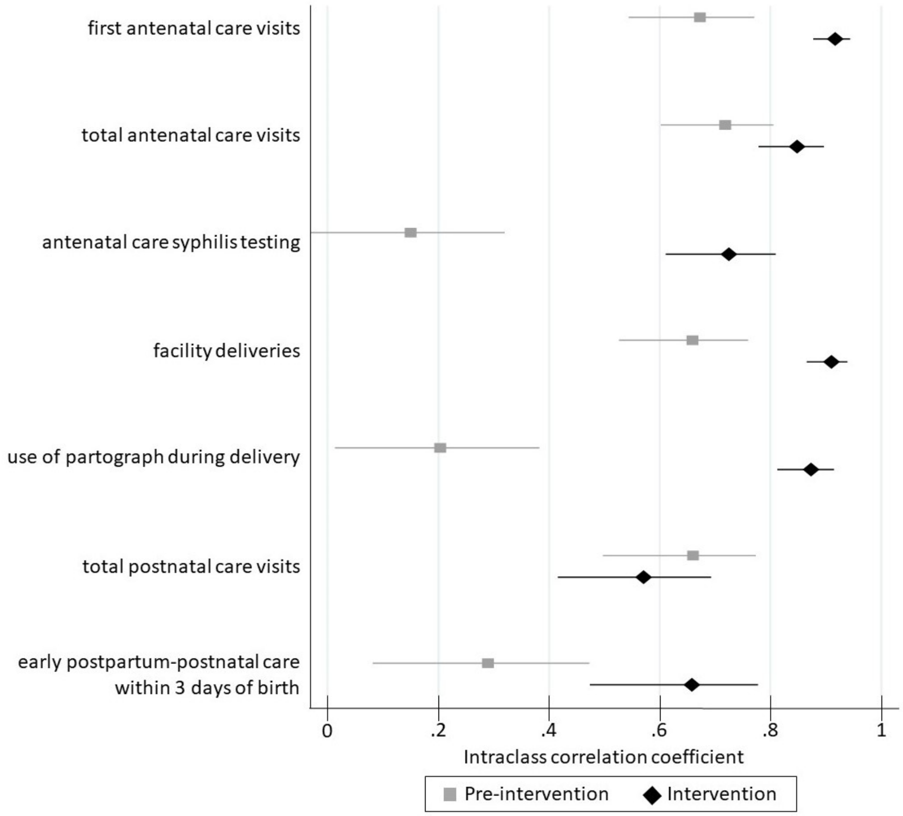

Figure 3. Accuracy of facility reporting: comparison of paper-based facility records and facility monthly reports in DHIS2 (n=97 facilities)
Notes: ICC values range from 0 to 1, with values approaching 1 representing greater reliability.

295x255mm (96 x 96 DPI)

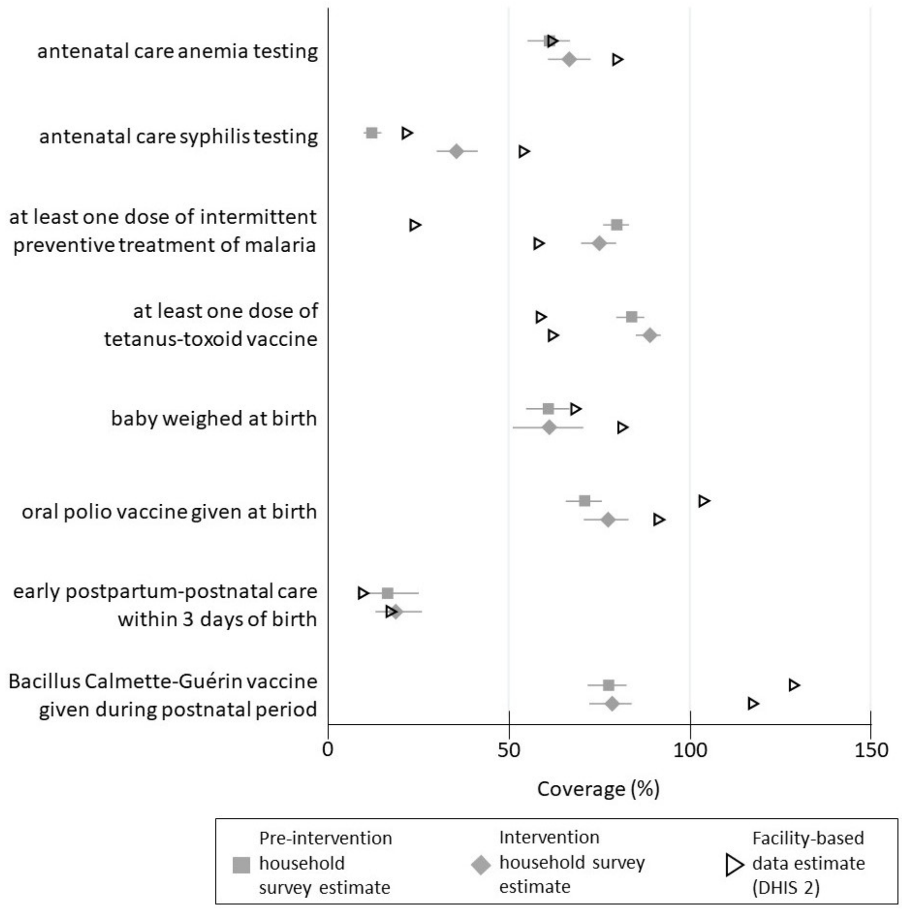

Figure 4. External consistency: comparison of household-level survey and facility summary reports in DHIS2 (n=97 facilities)

273x252mm (96 x 96 DPI)

Supplementary data

Table S1. Data quality metrics and data sources reviewed

Data quality metric	Analysis/calculation	Source(s)
**Data quality dimension 1: Completeness and timeliness**		
Facility-level metrics		
Availability of monthly facility reports	Proportion of facility's expected monthly reports actually submitted	facility monthly reports (DHIS2)
Timeliness of monthly facility reports	Proportion of facility's expected monthly reports actually submitted on time	facility monthly reports (DHIS2)
Completeness of all 14 priority maternal and newborn health data elements, per monthly facility report	Proportion of facility's submitted reports that have a value reported for all 14 priority maternal and newborn health data elements	facility monthly reports (DHIS2)
Indicator-level metrics		
Completeness of data element	Proportion of non-missing values for a given data element in expected monthly reports	facility monthly reports (DHIS2)
**Data quality dimension 2: Internal consistency**		
Consistency over time	Ratio of value of indicator for the intervention period to the mean of preceding 3 years	facility monthly reports (DHIS2)
Outliers	Number and frequency of moderate outliers (±2-3SD from the mean) and extreme outliers (±3SD from the mean) of monthly values	facility monthly reports (DHIS2)
Consistency between related data elements	Reliability index (intraclass correlation coefficient, ICC) comparing two sets of related data elements	facility monthly reports (DHIS2)
Accuracy of facility reporting (data accuracy, concordance)	Reliability index (intraclass correlation coefficient, ICC) comparing original facility register count to monthly facility report in DHIS2	facility registers; facility monthly reports (DHIS2)
**Data quality dimension 3: External consistency**		
Consistency between household surveys and monthly facility reports	Ratio of indicator values in household surveys for facility catchment areas to matching facilities in DHIS2	household surveys; facility monthly reports (DHIS2)

Notes:
DHIS2=District Health Information Software version 2, SD=standard deviation, ICC=intraclass correlation coefficient.

For peer review only

S2. Consistency over time, 2015-2018 (Gombe State, n=492 facilities)

Indicator, data element		2015	2016	2017	2018	Mean (2015-2017)	Ratio of 2018 to Mean (2015-2017)	Mean (2015-2016)	Ratio of Mean (2017-2018) to Mean (2015-2016)
Main denominators									
First antenatal care visits		108,136	131,263	132,715	128,924	124,038	1.04	119,700	1.09
Total antenatal care visits		194,141	365,251	351,596	300,404	303,663	0.99	279,696	1.17
Facility deliveries		59,731	48,335	65,211	52,122	57,759	0.90	54,033	1.09
Data elements									
Antenatal care anemia testing	Number	47,809	58,514	68,189	97,128	58,171	**1.67**	53,162	**1.55**
	Coverage	44%	45%	51%	75%	47%	**1.61**	44%	**1.43**
Antenatal care syphilis testing	Number	26,231	24,569	34,461	47,393	28,420	**1.67**	25,400	**1.61**
	Coverage	24%	19%	26%	37%	23%	**1.60**	21%	**1.46**
Iron-folic acid supplementation	Number	172,447	317,420	248,404	282,221	246,090	1.15	244,934	1.08
	Coverage	89%	87%	71%	94%	82%	1.14	88%	0.94
At least one dose of intermittent preventive treatment of malaria	Number	32,960	27,209	30,292	76,482	30,154	**2.54**	30,085	**1.77**
	Coverage	30%	21%	23%	59%	25%	**2.40**	26%	**1.60**
At least one dose of tetanus toxoid vaccine	Number	69,374	76,754	83,058	85,293	76,395	1.12	73,064	1.15
	Coverage	64%	58%	63%	66%	62%	1.07	61%	1.05
Delivery by skilled birth attendant	Number	18,912	10,240	32,725	47,042	20,626	**2.28**	14,576	**2.74**
	Coverage	32%	21%	50%	90%	34%	**2.63**	26%	**2.66**
Live births and still births	Number	33,492	39,892	47,715	49,053	40,366	1.22	36,692	1.32
	Coverage	56%	83%	73%	94%	70%	1.35	69%	1.21
Baby weighed at birth	Number	32,719	39,145	46,850	48,162	39,571	1.22	35,932	1.32
	Coverage	55%	81%	72%	92%	69%	**1.34**	68%	**1.21**
Oral polio vaccine at birth	Number	50,587	60,636	66,895	64,457	59,373	1.09	55,612	1.18
	Coverage	85%	125%	103%	124%	104%	1.19	105%	1.08
Early postpartum-postnatal care within 3 days of birth	Number	3,930	6,265	8,575	12,868	6,257	**2.06**	5,098	**2.10**
	Coverage	7%	13%	13%	25%	11%	**2.27**	10%	**1.94**
Bacillus Calmette-Guérin vaccine given during postnatal period	Number	75,491	84,670	87,525	81,362	82,562	0.99	80,081	1.05
	Coverage	126%	175%	134%	156%	145%	1.07	151%	0.96

For peer review only

S3. Presence of outliers in facility summary reports in DHIS2 in Gombe State, July 2015-December 2018 (n=492 facilities)

	July 2015-March 2017		April 2017-Dec 2018	
**Indicator, data element**	**Moderate Outliers**	**Extreme Outliers**	**Moderate Outliers**	**Extreme Outliers**
First antenatal visits	1 (Jan 2017)	0	1 (May 2018)	0
Total antenatal visits	1 (Oct 2016)	0	1 (May 2018)	0
Facility deliveries	0	0	1 (May 2018)	0
Antenatal care anemia testing	0	0	1 (May 2018)	0
Antenatal care syphilis testing	0	0	0	0
Iron-folic acid supplementation	0	1 (Oct 2016)	1 (May 2018)	0
At least one dose of intermittent preventive treatment of malaria	0	1 (Oct 2016)	0	0
At least one dose of tetanus toxoid vaccine	1 (Oct 2016)	0	1 (May 2018)	0
Delivery by skilled birth attendant	1 (Mar 2017)	0	1 (May 2018)	0
Live births and still births	1 (Mar 2017)	0	1 (May 2018)	0
Baby weighed at birth	1 (Mar 2017)	0	1 (May 2018)	0
Oral polio vaccine at birth	0	0	1 (May 2018)	0
Early postpartum-postnatal care within 3 days of birth	1 (Jan 2017)	0	0	0
Bacillus Calmette-Guérin vaccine given during postnatal period	1 (Jan 2017)	0	1 (May 2018)	0
**TOTAL OUTLIERS**	**8**<br>2 (Oct 2016),<br>3 (Jan 2017),<br>3 (Mar 2017)	**2**<br>2 (Oct 2016)	**11**<br>11 (May 2018)	**0**<br>0

For peer review only

STROBE Statement—checklist of items that should be included in reports of observational studies

	Item No	Recommendation	
**Title and abstract**	1	(*a*) Indicate the study's design with a commonly used term in the title or the abstract	Abstract Page 2
		(*b*) Provide in the abstract an informative and balanced summary of what was done and what was found	Page 2
**Introduction**			
Background/rationale	2	Explain the scientific background and rationale for the investigation being reported	Pages 3-4
Objectives	3	State specific objectives, including any prespecified hypotheses	Page 3
**Methods**			
Study design	4	Present key elements of study design early in the paper	Page 4
Setting	5	Describe the setting, locations, and relevant dates, including periods of recruitment, exposure, follow-up, and data collection	Pages 4-10
Participants	6	(*a*) *Cohort study*—Give the eligibility criteria, and the sources and methods of selection of participants. Describe methods of follow-up<br>*Case-control study*—Give the eligibility criteria, and the sources and methods of case ascertainment and control selection. Give the rationale for the choice of cases and controls<br>*Cross-sectional study*—Give the eligibility criteria, and the sources and methods of selection of participants	Pages 5-6
		(*b*) *Cohort study*—For matched studies, give matching criteria and number of exposed and unexposed<br>*Case-control study*—For matched studies, give matching criteria and the number of controls per case	
Variables	7	Clearly define all outcomes, exposures, predictors, potential confounders, and effect modifiers. Give diagnostic criteria, if applicable	Pages 8-10
Data sources/ measurement	8	For each variable of interest, give sources of data and details of methods of assessment (measurement). Describe comparability of assessment methods if there is more than one group	Pages 8-10
Bias	9	Describe any efforts to address potential sources of bias	Pages 8-10
Study size	10	Explain how the study size was arrived at	Page 5-6
Quantitative variables	11	Explain how quantitative variables were handled in the analyses. If applicable, describe which groupings were chosen and why	Pages 8-10
Statistical methods	12	(*a*) Describe all statistical methods, including those used to control for confounding	Pages 8-10
		(*b*) Describe any methods used to examine subgroups and interactions	n/a
		(*c*) Explain how missing data were addressed	Pages 8-10
		(*d*) *Cohort study*—If applicable, explain how loss to follow-up was addressed<br>*Case-control study*—If applicable, explain how matching of cases	n/a

For peer review only - http://bmjopen.bmj.com/site/about/guidelines.xhtml

		and controls was addressed<br>*Cross-sectional study*—If applicable, describe analytical methods taking account of sampling strategy	
		(*e*) Describe any sensitivity analyses	n/a
**Results**			
Participants	13	(a) Report numbers of individuals at each stage of study—eg numbers potentially eligible, examined for eligibility, confirmed eligible, included in the study, completing follow-up, and analysed	Page 10
		(b) Give reasons for non-participation at each stage	n/a
		(c) Consider use of a flow diagram	n/a
Descriptive data	14	(a) Give characteristics of study participants (eg demographic, clinical, social) and information on exposures and potential confounders	Pages 10-13
		(b) Indicate number of participants with missing data for each variable of interest	
		(c) *Cohort study*—Summarise follow-up time (eg, average and total amount)	
Outcome data	15	*Cohort study*—Report numbers of outcome events or summary measures over time	Pages 10-13
		Case-control study—Report numbers in each exposure category, or summary measures of exposure	
		Cross-sectional study—Report numbers of outcome events or summary measures	
Main results	16	(*a*) Give unadjusted estimates and, if applicable, confounder-adjusted estimates and their precision (eg, 95% confidence interval). Make clear which confounders were adjusted for and why they were included	Pages 10-13
		(*b*) Report category boundaries when continuous variables were categorized	n/a
		(*c*) If relevant, consider translating estimates of relative risk into absolute risk for a meaningful time period	n/a
Other analyses	17	Report other analyses done—eg analyses of subgroups and interactions, and sensitivity analyses	n/a
**Discussion**			
Key results	18	Summarise key results with reference to study objectives	Page 14
Limitations	19	Discuss limitations of the study, taking into account sources of potential bias or imprecision. Discuss both direction and magnitude of any potential bias	Pages 16-17
Interpretation	20	Give a cautious overall interpretation of results considering objectives, limitations, multiplicity of analyses, results from similar studies, and other relevant evidence	Pages 14-17
Generalisability	21	Discuss the generalisability (external validity) of the study results	Pages 16-17
**Other information**			
Funding	22	Give the source of funding and the role of the funders for the present study and, if applicable, for the original study on which the present article is based	Page 18

For peer review only

BMJ Open

Evaluating the quality of routine data captured in primary health facilities before and after an integrated district-focused intervention in Gombe State, northeastern Nigeria

Journal:	*BMJ Open*
Manuscript ID	bmjopen-2020-038174.R1
Article Type:	Original research
Date Submitted by the Author:	23-Jul-2020
Complete List of Authors:	Bhattacharya, Antoinette Alas; London School of Hygiene and Tropical Medicine Faculty of Infectious and Tropical Diseases, Department of Disease Control<br>Allen, Elizabeth; London School of Hygiene and Tropical Medicine Faculty of Epidemiology and Population Health, Department of Medical Statistics<br>Umar, Nasir; London School of Hygiene and Tropical Medicine Faculty of Infectious and Tropical Diseases, Disease Control<br>Audu, Ahmed; Gombe State Primary Health Care Development Agency<br>Felix, Habila; Gombe State Primary Health Care Development Agency<br>Schellenberg, Joanna; London School of Hygiene and Tropical Medicine, Disease Control<br>Marchant, Tanya; London School of Hygiene and Tropical Medicine Faculty of Infectious and Tropical Diseases, Disease Control
**Primary Subject Heading**:	Health services research
Secondary Subject Heading:	Health informatics
Keywords:	Health policy < HEALTH SERVICES ADMINISTRATION & MANAGEMENT, Maternal medicine < OBSTETRICS, PAEDIATRICS, Health informatics < BIOTECHNOLOGY & BIOINFORMATICS

SCHOLARONE™
Manuscripts

I, the Submitting Author has the right to grant and does grant on behalf of all authors of the Work (as defined in the below author licence), an exclusive licence and/or a non-exclusive licence for contributions from authors who are: i) UK Crown employees; ii) where BMJ has agreed a CC-BY licence shall apply, and/or iii) in accordance with the terms applicable for US Federal Government officers or employees acting as part of their official duties; on a worldwide, perpetual, irrevocable, royalty-free basis to BMJ Publishing Group Ltd ("BMJ") its licensees and where the relevant Journal is co-owned by BMJ to the co-owners of the Journal, to publish the Work in this journal and any other BMJ products and to exploit all rights, as set out in our licence.

The Submitting Author accepts and understands that any supply made under these terms is made by BMJ to the Submitting Author unless you are acting as an employee on behalf of your employer or a postgraduate student of an affiliated institution which is paying any applicable article publishing charge ("APC") for Open Access articles. Where the Submitting Author wishes to make the Work available on an Open Access basis (and intends to pay the relevant APC), the terms of reuse of such Open Access shall be governed by a Creative Commons licence – details of these licences and which Creative Commons licence will apply to this Work are set out in our licence referred to above.

Other than as permitted in any relevant BMJ Author's Self Archiving Policies, I confirm this Work has not been accepted for publication elsewhere, is not being considered for publication elsewhere and does not duplicate material already published. I confirm all authors consent to publication of this Work and authorise the granting of this licence.

For peer review only

Evaluating the quality of routine data captured in primary health facilities before and after an integrated district-focused intervention in Gombe State, northeastern Nigeria

Antoinette Alas Bhattacharya[1*], Elizabeth Allen[2], Nasir Umar[1], Ahmed Audu[3], Habila Felix[3], Joanna RM Schellenberg[1], and Tanya Marchant[1]

[1] Department of Disease Control, London School of Hygiene & Tropical Medicine, London, United Kingdom

[2] Department of Medical Statistics, London School of Hygiene & Tropical Medicine, London, United Kingdom

[3] State Primary Health Care Development Agency, Gombe, Nigeria

* Corresponding author

London School of Hygiene & Tropical Medicine

Keppel Street

London W1CE 7HT

United Kingdom

antoinette.bhattacharya@lshtm.ac.uk

+44 20 7636 8636

Word count: 3685

For peer review only - http://bmjopen.bmj.com/site/about/guidelines.xhtml

ABSTRACT

Objectives: Primary objective: to assess nine data quality metrics for 14 maternal and newborn health data elements, following implementation of an integrated, district-focused data quality intervention. Secondary objective: to consider whether assessing the data quality metrics beyond completeness and accuracy of facility reporting offered new insight into reviewing routine data quality.

Design: Before-and-after study design

Setting: Primary health facilities in Gombe State, northeastern Nigeria

Participants: Monitoring and evaluation officers and maternal, newborn, and child health coordinators for state-level and all 11 local government areas (district-equivalent) overseeing 492 primary care facilities offering maternal and newborn care services

Intervention: Between April 2017-December 2018, we implemented an integrated data quality intervention which included: introduction of job aids and regular self-assessment of data quality, peer review and feedback, learning workshops, work planning for improvement, and ongoing support through social media.

Outcome measures: 9 metrics for the data quality dimensions of completeness and timeliness, internal consistency of reported data, and external consistency

Results: The data quality intervention was associated with improvements in 7 of 9 data quality metrics assessed including availability and timeliness of reporting, completeness of data elements, accuracy of facility reporting, consistency between related data elements, and frequency of outliers reported. Improvement differed by data element type, with content of care and commodity-related data improving more than contact-related data. Increases in the consistency between related data elements demonstrated improved internal consistency within and across facility documentation.

Conclusions: An integrated district-focused data quality intervention – including regular self-assessment of data quality, peer review and feedback, learning workshops, work planning for improvement, and ongoing support through social media – can increase the completeness, accuracy, and internal consistency of facility-based routine data.

For peer review only - http://bmjopen.bmj.com/site/about/guidelines.xhtml

STRENGTHS AND LIMITATIONS OF THIS STUDY

- We extended the evidence on integrating data quality interventions within existing systems to improve the quality of facility-based data for monitoring and planning.
- We demonstrated the value of an integrated district-focused data quality intervention to include regular self-assessments of data quality, peer review and feedback, work planning for improvement, and ongoing support through social media.
- We assessed the usefulness of the World Health Organization's catalogue of data quality metrics to measure and monitor the quality of routine facility data, as data quality studies primarily review completeness and accuracy of facility reporting.
- Without a concurrent comparison group, our before-and-after analyses cannot eliminate the effects of concurrent events and activities on data quality metrics.

For peer review only

INTRODUCTION

Routine health information systems provide essential data for governments and stakeholders to make decisions for managing performance and optimizing service delivery.[1-3] Routine health information systems, which include facility-based data, have the potential to provide disaggregated statistics important for understanding disparities and inequities in the provision of quality services and related health outcomes.[4 5]

Effective use of routine health data is dependent, in part, on the quality of data.[1 4 6-9] Studies assessing the quality of routine health data have shown persistent challenges in incomplete and untimely reporting, incomplete indicator-level data, inaccurate facility reporting, and imprecise target population estimates for coverage.[10-13] Further, studies have noted considerably poorer data quality at the facility-level than at district-, state-, and national-levels, citing challenges in accurately capturing data as well as in tallying and summarizing service data for monthly reporting.[14-16]

For peer review only - http://bmjopen.bmj.com/site/about/guidelines.xhtml

Improving the quality of routine data is a priority given the potential of routine data to contribute to effective program monitoring and performance management. Efforts to improve the quality of routine data have included data quality checking with feedback as a low-resource and low-cost activity for strengthening data quality, particularly when implemented as a routine activity. Data quality checking with feedback have been shown to improve completeness, timeliness, and accuracy of facility reporting.[4 6 7 10 17 18] Knowledge transfer activities, such as mentoring, training, or workshops, have offered an opportunity to receive additional skills building in data quality checking or data use. [6 8 19-28] Knowledge sharing activities such as data review meetings and dashboards have brought together different health system-levels or peers within a health system level and include understanding the relative performance of teams on service coverage indicators or on dimensions of data quality.[6 8 20 22 29-31] The introduction of technology, either software such as DHIS 2 or a device to enhance data collection such as a tablet, has demonstrated improvements in completeness, timeliness, and error detection. However, these technology-based initiatives often required supervision, monitoring, and feedback to ensure errors were resolved and that data entered were accurate and consistent.[7 27 32 33] Activities which aligned with user priorities and were integrated within existing government systems were perceived to be advantageous as well as more likely to be adopted and adapted.[20]

Within the context of routine health information systems, the World Health Organization (WHO) has characterized routine data quality into four broad dimensions: completeness and timeliness; internal consistency; external consistency; and external comparisons.[34] While the abovementioned data quality interventions have demonstrated increases in completeness and accuracy of facility reporting[4 6 8 35 36], there are few peer-reviewed studies that quantitatively assessed changes in data quality metrics beyond this.[3 9 37]

In this study, our primary objective was to measure the changes in data quality metrics before and after the introduction of an integrated district-focused intervention in northeastern Nigeria for

For peer review only - http://bmjopen.bmj.com/site/about/guidelines.xhtml

routine facility data captured in primary health facilities. A secondary objective was to determine the extent to which expanding data quality metrics beyond completeness and accuracy of facility reporting offered new insight into reviewing data quality.

METHODS

Study design

This was a before-and-after study design for a data quality intervention in all 11 local government areas (LGA, district-equivalent) of Gombe State, northeastern Nigeria. We present results for the state (n=492 facilities), comparing the 21-month period before the intervention, July 2015-March 2017, with the 21-month period after introducing the intervention, April 2017-December 2018.

Study approvals

Study approvals were obtained from the London School of Hygiene & Tropical Medicine (reference 14091) and the Health Research Ethics Committees for Nigeria (reference NHREC/01/01/2007) and Gombe State (reference ADM/S/658/Vol. II/66).

Patient and public involvement

Patients or the public were not involved in the design, conduct, reporting, or dissemination of the research described here.

Study setting

For peer review only - http://bmjopen.bmj.com/site/about/guidelines.xhtml

Gombe State is located in northeastern Nigeria, a region with high maternal and newborn mortality at 1,549 per 100 000 live births and 35 per 1,000 live births, respectively.[38 39] With an estimated population of 2.9 million, Gombe is predominantly rural and 35%% of the women have some primary school education.[40 41] Most women access maternity care through public facilities. Seventy-two percent of women reported at least one antenatal care visit during their last pregnancy and 28% gave birth in a health facility.[39 41] In 2018, over 70% of facility deliveries took place in rural primary health facilities.[42]

Under Nigeria's national policy of *Primary Health Care Under One Roof*, the Gombe State Primary Health Care Development Agency oversees the administration and service delivery for primary health facilities across 11 local government areas (LGA); each LGA has 10-11 political wards (114 wards, total).[43 44] LGA monitoring and evaluation officers are responsible for community- and facility-level data collection, validation, and reporting to the state office. LGA maternal, neonatal, and child health (MNCH) coordinators support the supervision and implementation of services for women and children.

During the intervention period, Gombe State had 492 primary health facilities providing antenatal and childbirth services. As in other states in Nigeria, Gombe facility staff generally completed 13 paper-based registers to document the services they provide (Nigeria Health Management Information Systems, version 2013). Every month, a subset of data in these registers were tallied and summarized in a paper-based report and sent to the LGA health office to be entered into District Health Information Software, version 2 (DHIS2).

Data quality intervention

The routine data quality intervention period spanned 21 months, from April 2017 through December 2018. Following a situational analysis and literature review, development of the intervention took

For peer review only - http://bmjopen.bmj.com/site/about/guidelines.xhtml

place in consultation with the Gombe State Primary Health Care Development Agency. The design was predicated on three key issues. Interventions that include data quality assessments and feedback have demonstrated improvement in completeness, timeliness, and consistency.[4 6 8 19 20 22 23 26-28] Monitoring and evaluation officers and program coordinators in the state were expected to engage with data from facilities to support improvement of service delivery, though their interactions were not optimally structured to ensure efficient and complementary engagement with facility data. Further, the recent publication of the WHO data quality review toolkit in early 2017 provided clarity on assessing the data quality dimensions and metrics for facility data, though the Gombe district staff required further support and job aids to administer the data quality reviews as recommended in the WHO guidance.[45]

Thus, the intervention emphasized the partnership between the LGA monitoring and evaluation officer and the LGA maternal, newborn, and child health program coordinator to underscore the link between the quality and use of routine data.[8] The intervention was designed to facilitate existing LGA-level supervision responsibilities. It leveraged scheduling of ongoing activities to minimize cost, added job aids based on the WHO data quality review toolkit, and defined performance standards to provide structure to existing data quality checking duties and to target feedback to facilities.

The intervention included the following activities: (i) data quality learning workshops to present data quality self-assessment findings and develop work plans for improvement; (ii) defining data quality performance standards and milestones for completeness, timeliness, and consistency; (iii) introduction of job aids to self-assess data quality according to the WHO data quality metrics; (iv) monthly state- and LGA-level data quality summary reports; (v) intentional practice on providing constructive feedback to peers and low-performing facilities to promote a positive culture of data use; and (vi) ongoing engagement on data quality issues through government-approved communication channels, including the social media application WhatsApp.

For peer review only - http://bmjopen.bmj.com/site/about/guidelines.xhtml

Twenty-six main attendees participated in the workshops and ongoing communication in-between workshops. This included two participants from each of the 11 LGAs, a monitoring and evaluation officer and the maternal, newborn, and child health program coordinator. At the state-level, four officials participated: the director of the Gombe State Planning, Research, and Statistics Department, the state monitoring and evaluation officer, the state health management information systems officer, and the state maternal, newborn, and child health coordinator.

Four data quality learning workshops took place every six to nine months. The two-day workshops included the introduction of job aids and practical sessions to strengthen data quality checking skills, the presentation of the state's and each LGA's self-assessment of data quality, and the development of six-month work plans to improve the quality of facility-based routine data. Using materials designed for post-graduate learning and teaching, there was intentional practice on how to provide constructive feedback to peers and facilities to promote a positive culture of information use.[46]

At each workshop, a major theme emerged during the work planning sessions (Figure 1). For example, at the first workshop, participants were concerned with inconsistencies observed between the paper-based facility registers and the facility's monthly summary reports. Activities enacted from the work planning session were to revitalize dormant strategies previously set up to address program monitoring and evaluation activities: (i) LGA data validation committee meetings, where facilities bring their registers for verification against their submitted monthly facility report. and (ii) a social media WhatsApp group of LGA actors and facilities. The LGA teams posted pictures and comments on these facility interactions on the WhatsApp group for encouragement and accountability.

After the first workshop, the Gombe State monitoring and evaluation officer disseminated monthly state- and LGA-level data quality summary reports. LGAs were assessed according to the WHO data quality metrics and recommendations for improvement were offered. Initially, this activity was

For peer review only - http://bmjopen.bmj.com/site/about/guidelines.xhtml

designed for the external workshop facilitators to compose and disseminate, while building the capacity of the state officer to take on this task over time.

Outcomes

Using the WHO data quality review toolkit for routine facility data, we assessed 9 metrics across the three data quality dimensions of completeness and timeliness; internal consistency; and external consistency.[45] The data sources and analyses for each data quality metric are described in the following section. Supplementary table S1 provides additional information on each data quality metric assessed and the data sources reviewed.

Data analysis and data sources

Three data sources were used to assess the routine data quality metrics, described below: facility-reported data in DHIS2; external facility surveys; and external household surveys.

DHIS2 contained monthly reports for the 492 primary facilities providing antenatal care, childbirth, and postnatal care services. Monthly aggregated DHIS2 data for July 2015-December 2018 were downloaded at one time and included 14 maternal and newborn health-related data elements. These data were used to assess availability of facility reporting; timeliness of facility reporting; completeness of all 14 priority maternal and newborn health data elements, per monthly facility report; completeness of data element; presence of moderate and extreme outliers; consistency of indicator values over time; and consistency between related data elements.

To assess completeness of facility reporting, we compared the proportions of facilities submitting reports before the intervention and during the intervention. To assess timeliness of facility reporting, the numerator for completeness of facility reporting was further limited to facilities that submitted the reports by the given timelines. To assess both completeness of data-related metrics, we compared the proportions of facility reports where a value was present for the relevant data. To

For peer review only - http://bmjopen.bmj.com/site/about/guidelines.xhtml

compare the proportions of the pre-intervention and intervention periods, we used linear mixed models to account for the clustering of facility measurements within facilities and the clustering of facilities within districts. P-values for these metrics were computed from the Wald test within the mixed models. In these models, we also adjusted for potential confounders such as total client volume and time.[4 6]

We calculated the intraclass correlation coefficient (ICC) as a measure of agreement for the WHO metric assessing consistency between related data elements. ICC values range between 0 and 1, where values approaching 1 represented greater agreement between two related data elements. As the intervention worked through district-level staff to improve data quality across 492 primary health facilities, a paired t-test was carried out to compare pre-intervention and intervention periods matching ICCs at the district-level.

In 2016 and 2018, facility-level surveys were conducted in 97 primary facilities across Gombe to assess their readiness to provide maternal and newborn health services. The two surveys represented the approximate midpoints of the pre-intervention and intervention period. The selected facilities were a state-wide random sample drawn from all primary health facilities. Detailed methods are reported elsewhere.[47] The facility survey protocol was similar to a Service Availability and Readiness Assessment and also included data extraction from the facility's paper-based antenatal and postnatal care register and the labor and delivery register (Nigeria health management information system, version 2013).[48] A trained third party data collection team tallied and recorded the register data for each month of the six-month periods immediately prior to the survey: January-June 2016 and February-July 2018.

These data were used to assess the accuracy of facility reporting (also referred to as data accuracy, data verification, or concordance in peer-reviewed literature). We compared the facilities' paper-based registers data with the facilities' monthly reported data in DHIS2. As with the consistency between related data elements metric, we calculated the ICC as a measure of agreement and carried

For peer review only - http://bmjopen.bmj.com/site/about/guidelines.xhtml

out a paired t-test for the pre-intervention and intervention periods matching ICCs at the district level. ICC values approaching 1 represented greater agreement.

In 2016 and 2018, household-level surveys were conducted in the enumeration areas of the abovementioned 97 primary facilities to assess access to and quality of maternal and newborn services.[47] A total of 79 enumeration areas were surveyed since some enumeration areas were served by more than one facility. All households in each enumeration area were surveyed. The household surveys included a mother's module which asked all women who reported a birth in the last year a detailed set of questions about their contact with health services across the continuum of care from pregnancy to postnatal care. Informed consent was obtained at the community leadership-level and at the individual-level for each respondent. All invited participants agreed to be interviewed.

These data were used for external consistency during the pre-intervention and intervention periods. We compared coverage estimates from household surveys to those from the 97 matching facilities in DHIS2. We compared the same recall period for the surveys and the DHIS2. The DHIS2 data are considered consistent if they fall within the confidence intervals of the external household survey estimates. Calculations of point estimates and their 95% confidence intervals were done using the svyset Stata command (StataCorp, College Station, USA) to adjust for clustering. We chose the highest-order clustering level to provide the most conservative confidence interval estimates.[49]

RESULTS

An integrated district-focused data quality intervention was implemented across 11 LGAs overseeing 492 primary health facilities providing maternal and newborn care services. Below, we present the results for 9 data quality metrics.

For peer review only - http://bmjopen.bmj.com/site/about/guidelines.xhtml

Completeness and timeliness

Table 1 summarizes the completeness and timeliness of reporting at the facility- and indicator-levels.

At the facility-level, the availability of monthly facility reports improved from 72% to 82% (p<0.001) and timeliness of submitting the reports increased from 60% to 72% (p<0.001). The proportion facility-months where all 14 priority maternal and newborn health data elements contained a value within the monthly report increased from 62% to 68% (p<0.001).

At the indicator-level, seven of 14 data elements assessed improved in completeness compared to the pre-intervention period. Indicator-level completeness did not change for contact indicators such as first antenatal care visits, total antenatal care visits, and facility deliveries.

For peer review only

For peer review only - http://bmjopen.bmj.com/site/about/guidelines.xhtml

Table 1. Facility- and indicator-level completeness and timeliness, Gombe State (n=492 facilities)

	Pre-intervention Jul '15-Mar '17 % (95% CI)	Intervention Apr '17- Dec '18 % (95% CI)
**Facility-level**		
Availability of monthly facility reports	72 (69-74)	82 (80-84)
Timeliness of monthly facility reports	60 (57-62)	72 (70-74)
Completeness of all 14 priority maternal and newborn health data elements, per monthly facility report	62 (60-63)	68 (66-70)
**Indicator-level**		
For every 100 facilities that submitted a monthly facility report, the percentage of facilities reporting a value for the following services:		
First antenatal care visits	76 (67-85)	77 (70-84)
Total antenatal care visits	100 (99-100)	100 (100-100)
Facility deliveries	68 (59-77)	67 (60-74)
For every 100 facilities that reported a value for first ANC visit, the percentage of facilities reporting a value for the following services:		
Antenatal care anemia testing	28 (16-39)	36 (24-49)
Antenatal care syphilis testing	42 (23-61)	29 (23-35)*
Iron-folic acid supplementation	80 (75-84)	89 (85-92)
At least one dose administered of intermittent preventive treatment of malaria	45 (34-56)	56 (49-62)
At least one dose administered of tetanus toxoid vaccine	90 (86-93)	89 (86-91)
For every 100 facilities that reported a value for a facility delivery, the percentage of facilities reporting a value for the following services:		
Delivery by skilled birth attendant	43 (25-61)	86 (81-91)
Live birth or still birth	90 (86-95)	96 (94-97)
Baby weighed at birth	89 (83-95)	95 (94-97)
Oral polio vaccine given at birth	79 (70-87)	86 (82-90)
Early postpartum-postnatal care within 3 days of birth	45 (38-53)	55 (46-64)
Bacillus Calmette-Guérin vaccine given during postnatal care period	79 (71-88)	81 (77-86)

Notes:
* During the intervention period, commodities for antenatal care syphilis testing were redistributed and restricted to 57 facilities. For these 57 facilities, completeness of data for antenatal care syphilis testing increased from 48% (95% CI: 28-68) to 77% (95% CI: 69-86).

For peer review only - http://bmjopen.bmj.com/site/about/guidelines.xhtml

Internal consistency: consistency between related data elements

To assess the consistency between related data elements with a predictable relationship, two types of relationships were reviewed (Table 2). The first type of relationship assessed concurrent tallying across different data elements within and across facility registers. For example, (1) normal deliveries + caesarean deliveries + assisted deliveries = live births + still births; and (2) total postpartum visits reported = sum of the postnatal visit categories reported. For Gombe State, the ICC of delivery types (normal, caesarean, assisted) to birth types (live births, still births) improved from 0.83 (95%CI: 0.73-0.94) to 0.95 (0.93-0.97). Similar patterns of improvement were noted for postnatal visit tallying from an ICC of 0.46 (0.36-0.57) to 0.76 (0.66-0.85).

The second type of relationship assessed was a service provision compared to a contact indicator (e.g., the number of antenatal care syphilis testing done compared to antenatal care first visits, the number of babies weighed at birth compared to the number of facility deliveries). During the pre-intervention period, one of the 10 relationships reflected high consistency: iron-folic acid supplementation. During the implementation period, seven of the 10 relationships reflected improved consistency.

Internal consistency: accuracy of facility reporting

Comparing the facilities' registers with their submitted monthly reports, accuracy of facility reporting (data accuracy) had improved for 6 of 7 indicators, reflecting greater agreement during the intervention period (Table 3). For total antenatal care visits, there was considerable variation between districts during the pre-intervention and intervention periods, with an ICC of 0.62 (95% CI: 0.41-0.83) and 0.86 (95% CI 0.72-0.99), respectively.

For peer review only - http://bmjopen.bmj.com/site/about/guidelines.xhtml

Table 2. Internal consistency: consistency between data elements with a predictable relationship (n=492 facilities)

Data elements with a predictable relationship	ICC, Pre-intervention	ICC, Intervention	P-value
Concurrent tallying within and across facility documentation			
Normal + caesarean + assisted deliveries = livebirths + stillbirths	0.83 (0.73-0.94)	0.95 (0.93-0.98)	0.023
Total postnatal care visits = sum (postnatal care visit categories)	0.46 (0.36-0.57)	0.76 (0.66-0.85)	<0.001
Relationship between first antenatal care visits and:			
Antenatal care anemia testing	0.39 (0.18-0.59)	0.51 (0.35-0.68)	0.016
Antenatal care syphilis testing	0.19 (0.09-0.29)	0.49 (0.37-0.60)	0.003
At least one dose of intermittent preventive treatment of malaria	0.33 (0.17-0.48)	0.43 (0.32-0.54)	0.129
At least one dose of tetanus-toxoid vaccine	0.67 (0.53-0.80)	0.73 (0.65-0.82)	0.167
Relationship between total antenatal care visits and:			
Iron-folic acid supplementation	0.92 (0.89-0.95)	0.97 (0.95-0.99)	0.005
Relationship between facility deliveries and:			
Baby weighed at birth	0.82 (0.71-0.93)	0.94 (0.92-0.97)	<0.001
Delivery by skilled birth attendant	0.42 (0.26-0.59)	0.87 (0.80-0.94)	<0.001
Oral polio vaccine given at birth	0.48 (0.32-0.63)	0.72 (0.65-0.79)	0.007
Early postpartum-postnatal care within 3 days of birth	0.09 (0.06-0.13)	0.19 (0.13-0.24)	<0.001
Bacillus Calmette-Guérin vaccine given during postnatal period	0.56 (0.40-0.72)	0.67 (0.57-0.76)	0.147

Notes:
ICC values range from 0 to 1, with values approaching 1 representing greater agreement.

For peer review only - http://bmjopen.bmj.com/site/about/guidelines.xhtml

Table 3. Accuracy of facility reporting: comparison of paper-based facility records and facility monthly reports in DHIS2 (n=97 facilities)

Data element	ICC, Pre-intervention	ICC, Intervention	P-value
First antenatal care visits	0.57 (0.40-0.75)	0.88 (0.83-0.93)	0.004
Total antenatal care visits	0.62 (0.41-0.83)	0.86 (0.72-0.99)	0.050
Antenatal care syphilis testing	0.08 (-0.02-0.18)	0.59 (0.42-0.76)	<0.001
Facility deliveries	0.48 (0.26-0.70)	0.82 (0.69-0.94)	0.019
Use of partograph to monitor labor and delivery	0.24 (0.03-0.45)	0.83 (0.77-0.89)	<0.001
Total postnatal care visits	0.15 (0.02-0.29)	0.58 (0.38-0.78)	0.001
Early postpartum-postnatal care within 3 days of birth	0.21 (0.01-0.41)	0.55 (0.31-0.80)	0.020

Notes:
ICC values range from 0 to 1, with values approaching 1 representing greater agreement.

Internal consistency: outliers and consistency over time

Supplementary tables S2 and S3 summarize the presence of outliers and the consistency over time for the 14 maternal and newborn data elements. The frequency of months when outliers were reported decreased during the intervention period. However, 11 moderate outliers were reported during the intervention period compared to eight moderate outliers and two extreme outliers reported during the pre-intervention period. All 11 outliers reported during the intervention period occurred in May 2018 during a health worker strike. Of the 14 data elements assessed, six data elements were inconsistent over time due to reported increases in services when comparing the final year of the intervention 2018 to the mean value of the last 3 years and when comparing the pre-intervention and intervention periods.

For peer review only - http://bmjopen.bmj.com/site/about/guidelines.xhtml

External consistency: agreement between facility summary reports in DHIS2 and household surveys

Figure 2 summarizes external consistency, which is the agreement between facility-based routine data in DHIS2 compared to household-level surveys in the enumeration areas of these facilities. Other than the indicator for early postpartum-postnatal care, there was no agreement nor any consistent pattern of agreement between facility-based routine data and the household surveys. DHIS2 data underestimated compared to the household survey for at least one dose of intermittent preventative therapy for malaria in pregnancy and at least one dose of tetanus toxoid. DHIS2 data overestimated compared to the household survey for baby weighed at birth, oral polio vaccine given at birth, and Bacillus Calmette-Guérin given during postnatal period. For antenatal care anemia testing, facility-based estimates were within the household survey estimate confidence interval but overestimated compared to the household survey during the intervention period.

DISCUSSION

Facility-based routine data are an important source for monitoring, performance management, and planning.[9] Our study found that an integrated district-focused data quality intervention – which included regular self-assessment of data quality, peer review and feedback, learning workshops, work planning for improvement, monthly data quality reports, and ongoing support through social media – was associated with improvements across most WHO data quality metrics. There were differences in data quality improvement by data element type.[50] Data related to content of care or the provision of commodities, such as syphilis testing and intermittent preventative therapy for malaria, improved more across data quality metrics compared to contact indicators. Contact indicators had relatively higher data quality metrics before the intervention, such as first antenatal

For peer review only - http://bmjopen.bmj.com/site/about/guidelines.xhtml
For peer review only

care visits and facility deliveries. Contact indicators such as first antenatal care visits, fourth antenatal care visits, and facility deliveries are generally well-defined events to document, have been key denominators for local program planning, and have been prioritized for monitoring progress in previous global initiatives including the Millennium Development Goals and Countdown to 2015.[50-52]

This was an integrated data quality intervention designed to facilitate existing state- and district-level data quality checking responsibilities and emphasize the partnership between the monitoring and evaluation officers and the maternal, newborn and child health program coordinators to expand local access to the DHIS2 data, use the data, and problem solve.[9] Incorporating regular data quality reviews and feedback within supervision is an acknowledged low-cost and effective activity for improving completeness and consistency.[6 8 17 18 53] Leveraging existing staff and meeting schedules as well as adding structure to existing data-related responsibilities further promotes the feasibility of this intervention in similar settings. The evolution of the intervention through the work planning sessions prompted local solutions defined by the participants as feasible and within their resources to implement. In particular, the participants' decision to revitalize the data validation committees during the first workshop engaged the facilities early on to ensure the facility's register counts matched the facility's monthly report aggregate value. This early engagement with facilities could have contributed to the increased ICCs for accuracy of facility reporting observed during the intervention period (as shown in Table 3). A formative phase of the intervention might have captured activities such as the data validation committee as a pre-defined intervention activity. Including a formative phase should be a consideration for future implementation.

Our findings aligned with previous studies reporting improvements in completeness, timeliness, and accuracy of facility reporting after intervention. However, this study's relative gains may reflect the scale of working through 11 districts with 492 primary health facilities.[4 6 8 35 36] A data quality intervention in KwaZulu-Natal province, South Africa, which included trainings, monthly data

For peer review only - http://bmjopen.bmj.com/site/about/guidelines.xhtml

meetings, and external data quality audits across 78 facilities improved completeness of six data elements from 26% to 64% and the agreement between facility records and reports (data accuracy) from 37% to 65%.[6] A province-wide data quality intervention in Sofala, Mozambique, for 26 facilities included regular district-level review meetings for health workers and managers, data dashboards for tracking trends and rankings, human resource optimization models, and equipment purchase and maintenance. The summary measure used to evaluate data quality improvement, concordance, improved from 56% to 88% during the intervention period.[4] The introduction of an electronic medical record to support data quality improvement in 27 facilities across Kenya recorded a decline in missing data from 31% to 13% for 24 data elements, with a mean concordance score increasing across facilities by 1.79 (95% CI:0.25-3.33).[36]

While our findings align with previous studies for increased completeness, timeliness, and accuracy, our study reviewed additional WHO metrics to give a more comprehensive picture of the dimensions of data quality. This study also provided an opportunity to reflect on the relative usefulness of assessing all WHO data quality metrics to understand the quality of routine data in a given context. Sharp increases in service uptake due to health campaigns or targeted health projects make the assessment of moderate outliers and consistency over time less insightful about data quality, especially in the context of urgent efforts towards achieving universal health coverage. Our study found that, other than early postpartum-postnatal care within three days of birth, there were no instances of agreement between the facility-based routine data and external household surveys. However, an emerging body of criterion validity studies have demonstrated mixed results in the ability of women to recall facility-based pregnancy- and childbirth-related events in household surveys.[54-58] More research is needed on how to reconcile health facility and household survey data, while also reconsidering the emphasis on the household surveys as the reference standard.

The data quality metric to assess the consistency between related data elements provided useful insights in addition to the completeness and accuracy regularly reported in the literature. Assessing

For peer review only - http://bmjopen.bmj.com/site/about/guidelines.xhtml

the data relationship for a service provision compared to a contact indicator (e.g., ANC anemia testing compared to ANC first visits) allows for discussion on whether observed discrepancies are due to low service uptake or poor reporting, an important consideration given the emphasis on improving quality of care and understanding effective coverage.[59] Further, assessing data relationships that require concurrent tallying of services/information across data sources (e.g. facility attendance = inpatient + outpatient; normal delivery + caesarean delivery + assisted delivery = live births + still births), provides useful insight about whether a facility is paying attention to the internal consistency of their data within and across facility documentation. Focus on accuracy of facility reporting, a more common metric assessed in the peer-reviewed literature (referred to as data accuracy or concordance), is an important data quality metric as subnational-, national-, and global-level monitoring cannot take place effectively without the confidence that the facilities have summarized and tallied the data as intended. However, this focus on the accuracy of facility reporting up through the different levels of the health system do not require that these data be internally consistent with other data.

Our study had limitations. Without a concurrent comparison group, our before-and-after analyses cannot eliminate the effects of concurrent events and activities on data quality metrics. It is possible that other activities contributed to the observed data quality improvements. Given the high burden of maternal and neonatal mortality, the Gombe State Primary Health Care Development Agency spearheaded an initiative to improve maternal and neonatal services with the aim of having one fully functional primary health facility in each of its 114 wards. During the intervention period, 57 facilities (12% of the 492 maternity facilities) received support including facility-level quality improvement support as well as community-based outreach and education to increase uptake of services. Facility-level activities included support on data quality to monitor trends in services provided and the provision of computers and facility registers. Additionally, similar to other data quality assessments, we did not validate the data through direct clinical observations[4 6 10 12 32 60 61] nor did we compare the paper-based monthly summary reports to their electronic versions in DHIS2[6 12 37 62-64]. Despite close

For peer review only - http://bmjopen.bmj.com/site/about/guidelines.xhtml

attention to quality control, the facility- and household-level surveys might still be susceptible to errors in data recording, including incorrectly tallying the number of events in the original facility registers for comparison with data in DHIS2.

Improving the quality of routine facility data is essential for local and national evidence-based monitoring of universal health coverage. We found that an integrated district-focused data quality intervention was associated with increases across most WHO data quality metrics for routine facility-based data. Future initiatives should aim to incorporate national- and higher subnational-levels of the health system to determine scalability and sustainability of integrated data quality interventions in the long-term.

Acknowledgement

The authors wish to acknowledge the leadership of the Gombe State Primary Health Care Development Agency throughout the development and implementation of the data quality intervention. We also appreciate the cooperation of and partnership with the LGA M&E officers, MNCH coordinators, and Gombe State implementing partners to improve the quality of routine maternal and newborn health data in Gombe State. We are grateful to the women who participated in the household surveys and to the team who conducted the facility and household data collection. Finally, we are grateful to the facility teams responsible for providing and documenting their care for women and newborns.

Contributors

AAB and TM conceived and designed the study. AAB and EA designed the analyses. AAB carried out the analyses and composed the initial draft. JS, TM, EA, NU, AA, HF reviewed the early drafts. All authors approved the final draft.

For peer review only - http://bmjopen.bmj.com/site/about/guidelines.xhtml

Data sharing statement

Data for this study are available from the London School of Hygiene & Tropical Medicine public repository: http://datacompass.lshtm.ac.uk/229/.

Funding

This work was supported by the Bill & Melinda Gates Foundation [OPP1149259]. The funder of this study had no role in the study's design or conduct, data collection, analysis or interpretation of results, writing of the paper, or decision to submit for publication.

Competing interests

Mr Felix Habila and Mr Ahmed Audu are members of the Gombe State Primary Health Care Development Agency. The authors declare no other competing interests.

For peer review only

For peer review only - http://bmjopen.bmj.com/site/about/guidelines.xhtml

References

1. Nutley T. Improving data use in decision making: an intervention to strengthen health systems. Chapel Hill, North Carolina: MEASURE Evaluation, 2012.
2. AbouZahr C, Boerma T. Health information systems: the foundations of public health. *Bull World Health Organ* 2005;83(8):578-83.
3. Nisingizwe MP, Iyer HS, Gashayija M, et al. Toward utilization of data for program management and evaluation: quality assessment of five years of health management information system data in Rwanda. *Global Health Action* 2014;7:25829. doi: http://dx.doi.org/10.3402/gha.v7.25829
4. Wagenaar BH, Gimbel S, Hoek R, et al. Effects of a health information system data quality intervention on concordance in Mozambique: time-series analyses from 2009–2012. *Population Health Metrics* 2015;13(1):9. doi: 10.1186/s12963-015-0043-3
5. Chilundo B, Sundby J, Aanestad M. Analysing the quality of routine malaria data in Mozambique. *Malar J* 2004;3(3) doi: 10.1186/1475-2875-3-3
6. Mphatswe W, Mate KS, Bennett B, et al. Improving public health information: a data quality intervention in KwaZulu-Natal, South Africa. *Bull World Health Organ* 2012;90(3):176-82. doi: https://dx.doi.org/10.2471/BLT.11.092759
7. Mutale W, Chintu N, Amoroso C, et al. Improving health information systems for decision making across five sub-Saharan African countries: implementation strategies from the African Health Initiative. *BMC Health Serv Res* 2013;13(2):S9. doi: 10.1186/1472-6963-13-S2-S9
8. Braa J, Heywood A, Sahay S. Improving quality and use of data through data-use workshops: Zanzibar, United Republic of Tanzania. *Bull World Health Organ* 2012;90(5):379-84. doi: https://dx.doi.org/10.2471/BLT.11.099580
9. Maïga A, Jiwani SS, Mutua MK, et al. Generating statistics from health facility data: the state of routine health information systems in Eastern and Southern Africa. *BMJ Global Health* 2019;4(5):e001849. doi: 10.1136/bmjgh-2019-001849
10. Bosch-Capblanch X, Ronveaux O, Doyle V, et al. Accuracy and quality of immunization information systems in forty-one low income countries. *Trop Med Int Health* 2009;14(1):2-10. doi: 10.1111/j.1365-3156.2008.02181.x
11. Ronveaux O, Rickert D, Hadler S, et al. The immunization data quality audit: verifying the quality and consistency of immunization monitoring systems. *Bull World Health Organ* 2005;83(7):503-10.
12. Mate KS, Bennett B, Mphatswe W, et al. Challenges for routine health system data management in a large public program to prevent mother-to-child HIV transmission in South Africa. *PLoS One* 2009;4(5):e5483.
13. Maina I, Wanjala P, Soti D, et al. Using health-facility data to assess subnational coverage of maternal and child health indicators, Kenya. *Bull World Health Organ* 2017;95(10):683-94. doi: 10.2471/BLT.17.194399
14. Belay H, Lippeveld T. Inventory of PRISM framework and tools: application of PRISM tools and interventions for strengthening routine health information system performance. Working Paper Series No: WP-13-138. Chapel Hill, North Carolina, USA: MEASURE Evaluation, 2013.
15. Hotchkiss DR, Aqil A, Lippeveld T, et al. Evaluation of the Performance of Routine Information System Management (PRISM) framework: evidence from Uganda. *BMC Health Serv Res* 2010;10:188.
16. Aqil A, Avila JE, Mejia LSP, et al. Guanajuato SINAIS assessment. Technical Report No: SR-10-60. Chapel Hill, North Carolina, USA: MEASURE Evaluation, 2010.
17. Lippeveld T, Sauerborn R, Bodart C, et al. Design and implementation of health information systems. Geneva: World Health Organization 2000.

For peer review only - http://bmjopen.bmj.com/site/about/guidelines.xhtml

18. Ledikwe JH, Grignon J, Lebelonyane R, et al. Improving the quality of health information: a qualitative assessment of data management and reporting systems in Botswana. *Health Res Policy Syst* 2014;12:7.
19. Admon AJ, Bazile J, Makungwa H, et al. Assessing and improving data quality from community health workers: a successful intervention in Neno, Malawi. *Public Health Action* 2013;3 doi: 10.5588/pha.12.0071
20. Gimbel S, Mwanza M, Nisingizwe MP, et al. Improving data quality across 3 sub-Saharan African countries using the Consolidated Framework for Implementation Research: results from the African Health Initiative. *BMC Health Serv Res* 2017;17(Suppl 3):828. doi: 10.1186/s12913-017-2660-y
21. Karemere JN. Centers of excellence in monitoring and evaluation: an approach to improving data quality for effective decision making in the Democratic Republic of the Congo. *Am J Trop Med Hyg* 2017;97(5 Supplement 1):284-85.
22. Orji B, Olisaekee G, Udenze O, et al. Improving quality of data to advance malaria in pregnancy indicator coverage in Ebonyi State, Nigeria. *Am J Trop Med Hyg* 2017;97(5 Supplement 1):335.
23. Westercamp N, Staedke S, Hutchinson E, et al. Effectiveness and sustainability of a collaborative improvement method to increase the quality of routine malaria surveillance data in Kayunga District, Uganda. *Am J Trop Med Hyg* 2017;97(5 Supplement 1):31.
24. Kamadjeu RM, Tapang EM, Moluh RN. Designing and implementing an electronic health record system in primary care practice in sub-Saharan Africa: a case study from Cameroon. *Inform Prim Care* 2005;13(3):179-86.
25. Barrington J, Wereko-Brobby O, Ward P, et al. SMS for Life: a pilot project to improve anti-malarial drug supply management in rural Tanzania using standard technology. *Malar J* 2010;9 doi: 10.1186/1475-2875-9-298
26. Ledikwe JH, Reason LL, Burnett SM, et al. Establishing a health information workforce: innovation for low- and middle-income countries. *Human Resources for Health* 2013;11:35. doi: https://dx.doi.org/10.1186/1478-4491-11-35
27. Kiberu VM, Matovu JKB, Makumbi F, et al. Strengthening district-based health reporting through the district health management information software system: the Ugandan experience. *BMC Med Inform Decis Mak* 2014;14(1):40. doi: 10.1186/1472-6947-14-40
28. Ye M, N'Gbichi JM, Franchard T, et al. Malaria data quality and use in selected centers of excellence in Madagascar: results from a cross-sectional baseline survey. *Am J Trop Med Hyg* 2019;101(5 Supplement):118-19. doi: http://dx.doi.org/10.4269/ajtmh.abstract2019
29. Sherr K, Cuembelo F, Michel C, et al. Strengthening integrated primary health care in Sofala, Mozambique. *BMC Health Serv Res* 2013;13 Suppl 2:S4. doi: https://dx.doi.org/10.1186/1472-6963-13-S2-S4
30. Dodo M, Badolo O, Nebie S, et al. Contribution of quarterly malaria data review and validation to data quality and malaria services improvement. *Am J Trop Med Hyg* 2018;99(4 Supplement):118.
31. Tuti T, Bitok M, Malla L, et al. Improving documentation of clinical care within a clinical information network: an essential initial step in efforts to understand and improve care in Kenyan hospitals. *BMJ Global Health* 2016;1(1):e000028. doi: 10.1136/bmjgh-2016-000028
32. Puttkammer N, Baseman JG, Devine EB, et al. An assessment of data quality in a multi-site electronic medical record system in Haiti. *Int J Med Inform* 2016;86:104-16.
33. Maokola W, Willey BA, Shirima K, et al. Enhancing the routine health information system in rural southern Tanzania: successes, challenges and lessons learned. *Trop Med Int Health* 2011;16(6):721-30.
34. MEASURE Evaluation. Routine health information systems: a curriculum on basic concepts and practices. Chapel Hill, North Carolina, USA: MEASURE Evaluation, 2017.

For peer review only - http://bmjopen.bmj.com/site/about/guidelines.xhtml

35. Gething P, Noor A, Gikandi P, et al. Improving imperfect data from health management information systems in Africa using space-time geostatistics. *PLoS Med* 2006;3 doi: 10.1371/journal.pmed.0030271
36. Muthee V, Bochner AF, Osterman A, et al. The impact of routine data quality assessments on electronic medical record data quality in Kenya. *PLoS One* 2018;13(4):e0195362. doi: https://dx.doi.org/10.1371/journal.pone.0195362
37. Gimbel S, Micek M, Lambdin B, et al. An assessment of routine primary care health information system data quality in Sofala Province, Mozambique. *Population Health Metrics* 2011;9:12.
38. Izugbara CO, Wekesah FM, Adedini SA. Maternal health in Nigeria: a situation update. Nairobi, Kenya: African Population and Health Research Center, 2016.
39. National Bureau of Statistics and United Nations Children's Fund. Multiple Indicator Cluster Survey 2016-17: Survey Findings Report. Abuja, Nigeria: National Bureau of Statistics and United Nations Children's Fund, 2017.
40. Gombe State Government. History of Gombe State, Nigeria 2018 [Available from: http://gombestate.gov.ng/history-2/ accessed September 9, 2019.
41. National Population Commission [Nigeria] and ICF International. Nigeria Demographic and Health Survey 2018, Final Report. Abuja, Nigeria, and Rockville, Maryland, USA: NPC/Nigeria and ICF International, 2019.
42. Nigeria District Health Information System version 2. Nigeria Health Management Information System, version 2013: Gombe State facility deliveries, by facility type, January-December 2018. Abuja: Nigeria District Health Information System, version 2, 2019.
43. Federal Government of Nigeria. Integrating primary healthcare governance in Nigeria: implementation manual, 2013.
44. National Primary Health Care Development Agency. Integrating primary health care governance in Nigeria (Primary Health Care Under One Roof). Abuja: NPHCDA, 2013.
45. World Health Organization. Data quality review: a toolkit for facility data quality assessment. Geneva: World Health Organization, 2017.
46. Lippeveld T. Routine health facility and community information systems: creating an information use culture. *Global Health: Science and Practice* 2017;5(3):338–40.
47. Marchant T. Change in maternal and newborn health care. Interactions between families and frontline workers – their frequency, quality, and equity – and coverage of interventions for mothers and newborns. Report from Gombe State, Nigeria, 2012-2015. IDEAS Project Report: London School of Hygiene and Tropical Medicine, 2016.
48. World Health Organization. Service Availability and Readiness Assessment (SARA): an annual monitoring system for service delivery. Reference Manual, Version 2.2. Geneva: World Health Organization, 2015.
49. Bottomley C, Kirby MJ, Lindsay SW, et al. Can the buck always be passed to the highest level of clustering? *BMC Med Res Methodol* 2016;16:29-29. doi: 10.1186/s12874-016-0127-1
50. Bhattacharya AA, Umar N, Audu A, et al. Quality of routine facility data for monitoring priority maternal and newborn indicators in DHIS2: a case study from Gombe State, Nigeria. *PLoS One* 2019;14(1):e0211265. doi: https://dx.doi.org/10.1371/journal.pone.0211265
51. Bryce J, Requejo J, Countdown to 2015 Working Group. Countdown to 2015: tracking progress in maternal, newborn, and child survival. The 2008 report. New York, USA: UNICEF, 2008.
52. World Health Organization. Countdown to 2015: a decade of tracking progress for maternal, newborn and child survival, final report. Geneva: World Health Organization, 2015.
53. Wagenaar BH, Hirschhorn LR, Henley C, et al. Data-driven quality improvement in low-and middle-income country health systems: lessons from seven years of implementation experience across Mozambique, Rwanda, and Zambia. *BMC Health Serv Res* 2017;17 doi: 10.1186/s12913-017-2661-x
54. Stanton CK, Rawlins B, Drake M, et al. Measuring coverage in MNCH: testing the validity of women's self-report of key maternal and newborn health interventions during the

For peer review only - http://bmjopen.bmj.com/site/about/guidelines.xhtml

peripartum period in Mozambique. *PLoS One* 2013;8(5):e60694. doi: 10.1371/journal.pone.0060694
55. Blanc AK, Warren C, McCarthy KJ, et al. Assessing the validity of indicators of the quality of maternal and newborn health care in Kenya. *Journal of Global Health* 2016;6(1):010405. doi: 10.7189/jogh.06.010405
56. Blanc AK, Diaz C, McCarthy KJ, et al. Measuring progress in maternal and newborn health care in Mexico: validating indicators of health system contact and quality of care. *BMC Pregnancy Childbirth* 2016;16(1):255. doi: 10.1186/s12884-016-1047-0
57. McCarthy KJ, Blanc AK, Warren CE, et al. Women's recall of maternal and newborn interventions received in the postnatal period: a validity study in Kenya and Swaziland. *Journal of Global Health* 2018;8(1):010605. doi: 10.7189/jogh.08.010605 [published Online First: 2018/05/12]
58. Bhattacharya AA, Allen E, Umar N, et al. Monitoring childbirth care in primary health facilities: a validity study in Gombe State, northeastern Nigeria. *Journal of Global Health* 2019;9(2):020411. doi: 10.7189/jogh.09.020411
59. Amouzou A, Leslie HH, Ram M, et al. Advances in the measurement of coverage for RMNCH and nutrition: from contact to effective coverage. *BMJ Global Health* 2019;4(Suppl 4):e001297. doi: 10.1136/bmjgh-2018-001297
60. Glèlè Ahanhanzo Y, Ouendo E-M, Kpozehouen A, et al. Data quality assessment in the routine health information system: an application of the lot quality assurance sampling in Benin. *Health Policy Plan* 2015;30(7):837-43.
61. Makombe SD, Hochgesang M, Jahn A, et al. Assessing the quality of data aggregated by antiretroviral treatment clinics in Malawi. *Bull World Health Organ* 2008;86(4):310-4.
62. Nicol E, Dudley L, Bradshaw D. Assessing the quality of routine data for the prevention of mother-to-child transmission of HIV: an analytical observational study in two health districts with high HIV prevalence in South Africa. *Int J Med Inform* 2016;95:60-70.
63. Mlambo MG, Peltzer K, Koivu A. Prevention of mother-to-child transmission of HIV data completeness and accuracy assessment in health facilities of the Nkangala District. *Health SA Gesondheid* 2014;19(1):a774.
64. Omoleke SA, Tadesse MG. A pilot study of routine immunization data quality in Bunza Local Government area: causes and possible remedies. *The Pan African Medical Journal* 2017;27:239. doi: https://dx.doi.org/10.11604/pamj.2017.27.239.11875

For peer review only - http://bmjopen.bmj.com/site/about/guidelines.xhtml

Figures

Figure 1. Data quality learning workshops in Gombe State, April 2017-December 2018

Figure 2. External consistency: comparison of household-level survey and facility summary reports in DHIS2 (n=97 facilities)

For peer review only

For peer review only - http://bmjopen.bmj.com/site/about/guidelines.xhtml

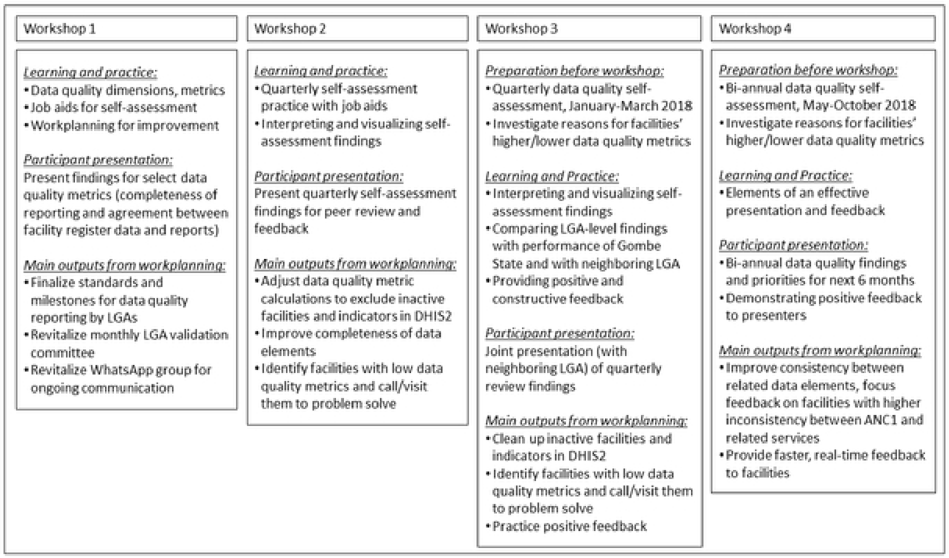

Figure 1. Data quality learning workshops in Gombe State, April 2017-December 2018

54x30mm (300 x 300 DPI)

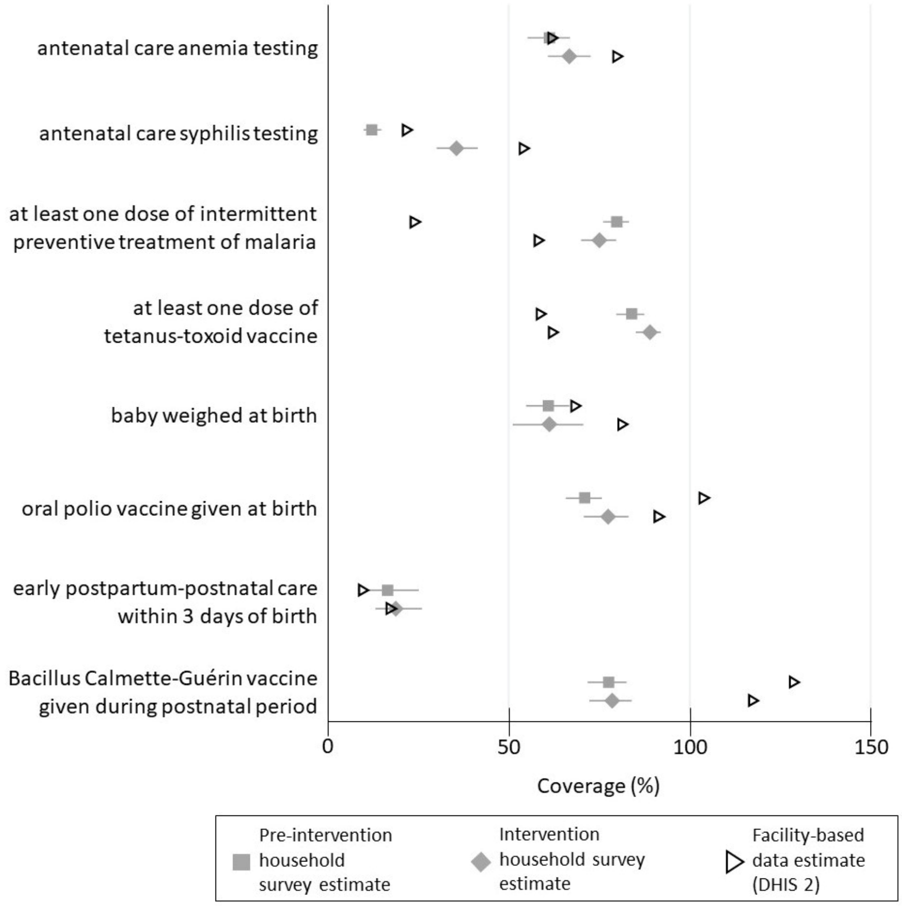

Figure 2. External consistency: comparison of household-level survey and facility summary reports in DHIS2 (n=97 facilities)

87x80mm (300 x 300 DPI)

Supplementary data

Table S1. Data quality metrics and data sources reviewed

Data quality metric	Analysis/calculation	Source(s)
**Data quality dimension 1: Completeness and timeliness**		
Facility-level metrics		
Availability of monthly facility reports	Proportion of facility's expected monthly reports actually submitted	facility monthly reports (DHIS2)
Timeliness of monthly facility reports	Proportion of facility's expected monthly reports actually submitted on time	facility monthly reports (DHIS2)
Completeness of all 14 priority maternal and newborn health data elements, per monthly facility report	Proportion of facility's submitted reports that have a value reported for all 14 priority maternal and newborn health data elements	facility monthly reports (DHIS2)
Indicator-level metrics		
Completeness of data element	Proportion of non-missing values for a given data element in expected monthly reports	facility monthly reports (DHIS2)
**Data quality dimension 2: Internal consistency**		
Consistency over time	Ratio of value of indicator for the intervention period to the mean of preceding 3 years	facility monthly reports (DHIS2)
Outliers	Number and frequency of moderate outliers ($\pm$2-3SD from the mean) and extreme outliers ($\pm$3SD from the mean) of monthly values	facility monthly reports (DHIS2)
Consistency between related data elements	Reliability index (intraclass correlation coefficient, ICC) comparing two sets of related data elements	facility monthly reports (DHIS2)
Accuracy of facility reporting (data accuracy, concordance)	Reliability index (intraclass correlation coefficient, ICC) comparing original facility register count to monthly facility report in DHIS2	facility registers; facility monthly reports (DHIS2)
**Data quality dimension 3: External consistency**		
Consistency between household surveys and monthly facility reports	Ratio of indicator values in household surveys for facility catchment areas to matching facilities in DHIS2	household surveys; facility monthly reports (DHIS2)

Notes:
DHIS2=District Health Information Software version 2, SD=standard deviation, ICC=intraclass correlation coefficient.

For peer review only

S2. Consistency over time, 2015-2018 (Gombe State, n=492 facilities)

Indicator, data element		2015	2016	2017	2018	Mean (2015-2017)	Ratio of 2018 to Mean (2015-2017)	Mean (2015-2016)	Ratio of Mean (2017-2018) to Mean (2015-2016)
Main denominators									
First antenatal care visits		108,136	131,263	132,715	128,924	124,038	1.04	119,700	1.09
Total antenatal care visits		194,141	365,251	351,596	300,404	303,663	0.99	279,696	1.17
Facility deliveries		59,731	48,335	65,211	52,122	57,759	0.90	54,033	1.09
Data elements									
Antenatal care anemia testing	Number	47,809	58,514	68,189	97,128	58,171	**1.67**	53,162	**1.55**
	Coverage	44%	45%	51%	75%	47%	**1.61**	44%	**1.43**
Antenatal care syphilis testing	Number	26,231	24,569	34,461	47,393	28,420	**1.67**	25,400	**1.61**
	Coverage	24%	19%	26%	37%	23%	**1.60**	21%	**1.46**
Iron-folic acid supplementation	Number	172,447	317,420	248,404	282,221	246,090	1.15	244,934	1.08
	Coverage	89%	87%	71%	94%	82%	1.14	88%	0.94
At least one dose of intermittent preventive treatment of malaria	Number	32,960	27,209	30,292	76,482	30,154	**2.54**	30,085	**1.77**
	Coverage	30%	21%	23%	59%	25%	**2.40**	26%	**1.60**
At least one dose of tetanus toxoid vaccine	Number	69,374	76,754	83,058	85,293	76,395	1.12	73,064	1.15
	Coverage	64%	58%	63%	66%	62%	1.07	61%	1.05
Delivery by skilled birth attendant	Number	18,912	10,240	32,725	47,042	20,626	**2.28**	14,576	**2.74**
	Coverage	32%	21%	50%	90%	34%	**2.63**	26%	**2.66**
Live births and still births	Number	33,492	39,892	47,715	49,053	40,366	1.22	36,692	1.32
	Coverage	56%	83%	73%	94%	70%	1.35	69%	1.21
Baby weighed at birth	Number	32,719	39,145	46,850	48,162	39,571	1.22	35,932	1.32
	Coverage	55%	81%	72%	92%	69%	**1.34**	68%	**1.21**
Oral polio vaccine at birth	Number	50,587	60,636	66,895	64,457	59,373	1.09	55,612	1.18
	Coverage	85%	125%	103%	124%	104%	1.19	105%	1.08
Early postpartum-postnatal care within 3 days of birth	Number	3,930	6,265	8,575	12,868	6,257	**2.06**	5,098	**2.10**
	Coverage	7%	13%	13%	25%	11%	**2.27**	10%	**1.94**
Bacillus Calmette-Guérin vaccine given during postnatal period	Number	75,491	84,670	87,525	81,362	82,562	0.99	80,081	1.05
	Coverage	126%	175%	134%	156%	145%	1.07	151%	0.96

For peer review only

S3. Presence of outliers in facility summary reports in DHIS2 in Gombe State, July 2015-December 2018 (n=492 facilities)

	July 2015-March 2017		April 2017-Dec 2018	
**Indicator, data element**	**Moderate Outliers**	**Extreme Outliers**	**Moderate Outliers**	**Extreme Outliers**
First antenatal visits	1 (Jan 2017)	0	1 (May 2018)	0
Total antenatal visits	1 (Oct 2016)	0	1 (May 2018)	0
Facility deliveries	0	0	1 (May 2018)	0
Antenatal care anemia testing	0	0	1 (May 2018)	0
Antenatal care syphilis testing	0	0	0	0
Iron-folic acid supplementation	0	1 (Oct 2016)	1 (May 2018)	0
At least one dose of intermittent preventive treatment of malaria	0	1 (Oct 2016)	0	0
At least one dose of tetanus toxoid vaccine	1 (Oct 2016)	0	1 (May 2018)	0
Delivery by skilled birth attendant	1 (Mar 2017)	0	1 (May 2018)	0
Live births and still births	1 (Mar 2017)	0	1 (May 2018)	0
Baby weighed at birth	1 (Mar 2017)	0	1 (May 2018)	0
Oral polio vaccine at birth	0	0	1 (May 2018)	0
Early postpartum-postnatal care within 3 days of birth	1 (Jan 2017)	0	0	0
Bacillus Calmette-Guérin vaccine given during postnatal period	1 (Jan 2017)	0	1 (May 2018)	0
**TOTAL OUTLIERS**	**8**<br>2 (Oct 2016),<br>3 (Jan 2017),<br>3 (Mar 2017)	**2**<br>2 (Oct 2016)	**11**<br>11 (May 2018)	**0**<br>0

BMJ Open

Improving the quality of routine maternal and newborn data captured in primary health facilities in Gombe State, northeastern Nigeria: a before-and-after study

Journal:	*BMJ Open*
Manuscript ID	bmjopen-2020-038174.R2
Article Type:	Original research
Date Submitted by the Author:	15-Oct-2020
Complete List of Authors:	Bhattacharya, Antoinette Alas; London School of Hygiene and Tropical Medicine Faculty of Infectious and Tropical Diseases, Department of Disease Control<br>Allen, Elizabeth; London School of Hygiene and Tropical Medicine Faculty of Epidemiology and Population Health, Department of Medical Statistics<br>Umar, Nasir; London School of Hygiene and Tropical Medicine Faculty of Infectious and Tropical Diseases, Disease Control<br>Audu, Ahmed; Gombe State Primary Health Care Development Agency<br>Felix, Habila; Gombe State Primary Health Care Development Agency<br>Schellenberg, Joanna; London School of Hygiene and Tropical Medicine, Disease Control<br>Marchant, Tanya; London School of Hygiene and Tropical Medicine Faculty of Infectious and Tropical Diseases, Disease Control
**Primary Subject Heading**:	Health services research
Secondary Subject Heading:	Health informatics, Public health
Keywords:	Health policy < HEALTH SERVICES ADMINISTRATION & MANAGEMENT, Maternal medicine < OBSTETRICS, PAEDIATRICS, Health informatics < BIOTECHNOLOGY & BIOINFORMATICS

SCHOLARONE™
Manuscripts

I, the Submitting Author has the right to grant and does grant on behalf of all authors of the Work (as defined in the below author licence), an exclusive licence and/or a non-exclusive licence for contributions from authors who are: i) UK Crown employees; ii) where BMJ has agreed a CC-BY licence shall apply, and/or iii) in accordance with the terms applicable for US Federal Government officers or employees acting as part of their official duties; on a worldwide, perpetual, irrevocable, royalty-free basis to BMJ Publishing Group Ltd ("BMJ") its licensees and where the relevant Journal is co-owned by BMJ to the co-owners of the Journal, to publish the Work in this journal and any other BMJ products and to exploit all rights, as set out in our licence.

The Submitting Author accepts and understands that any supply made under these terms is made by BMJ to the Submitting Author unless you are acting as an employee on behalf of your employer or a postgraduate student of an affiliated institution which is paying any applicable article publishing charge ("APC") for Open Access articles. Where the Submitting Author wishes to make the Work available on an Open Access basis (and intends to pay the relevant APC), the terms of reuse of such Open Access shall be governed by a Creative Commons licence – details of these licences and which Creative Commons licence will apply to this Work are set out in our licence referred to above.

Other than as permitted in any relevant BMJ Author's Self Archiving Policies, I confirm this Work has not been accepted for publication elsewhere, is not being considered for publication elsewhere and does not duplicate material already published. I confirm all authors consent to publication of this Work and authorise the granting of this licence.

For peer review only

Improving the quality of routine maternal and newborn data captured in primary health facilities in Gombe State, northeastern Nigeria: a before-and-after study

Antoinette Alas Bhattacharya[1*], Elizabeth Allen[2], Nasir Umar[1], Ahmed Audu[3], Habila Felix[3], Joanna RM Schellenberg[1], and Tanya Marchant[1]

[1] Department of Disease Control, London School of Hygiene & Tropical Medicine, London, United Kingdom

[2] Department of Medical Statistics, London School of Hygiene & Tropical Medicine, London, United Kingdom

[3] State Primary Health Care Development Agency, Gombe, Nigeria

* Corresponding author

London School of Hygiene & Tropical Medicine

Keppel Street

London W1CE 7HT

United Kingdom

antoinette.bhattacharya@lshtm.ac.uk

+44 20 7636 8636

Word count: 3685

For peer review only - http://bmjopen.bmj.com/site/about/guidelines.xhtml

ABSTRACT

Objectives: Primary objective: to assess nine data quality metrics for 14 maternal and newborn health data elements, following implementation of an integrated, district-focused data quality intervention. Secondary objective: to consider whether assessing the data quality metrics beyond completeness and accuracy of facility reporting offered new insight into reviewing routine data quality.

Design: Before-and-after study design

Setting: Primary health facilities in Gombe State, northeastern Nigeria

Participants: Monitoring and evaluation officers and maternal, newborn, and child health coordinators for state-level and all 11 local government areas (district-equivalent) overseeing 492 primary care facilities offering maternal and newborn care services

Intervention: Between April 2017-December 2018, we implemented an integrated data quality intervention which included: introduction of job aids and regular self-assessment of data quality, peer review and feedback, learning workshops, work planning for improvement, and ongoing support through social media.

Outcome measures: 9 metrics for the data quality dimensions of completeness and timeliness, internal consistency of reported data, and external consistency

Results: The data quality intervention was associated with improvements in 7 of 9 data quality metrics assessed including availability and timeliness of reporting, completeness of data elements, accuracy of facility reporting, consistency between related data elements, and frequency of outliers reported. Improvement differed by data element type, with content of care and commodity-related data improving more than contact-related data. Increases in the consistency between related data elements demonstrated improved internal consistency within and across facility documentation.

Conclusions: An integrated district-focused data quality intervention – including regular self-assessment of data quality, peer review and feedback, learning workshops, work planning for improvement, and ongoing support through social media – can increase the completeness, accuracy, and internal consistency of facility-based routine data.

For peer review only - http://bmjopen.bmj.com/site/about/guidelines.xhtml

STRENGTHS AND LIMITATIONS OF THIS STUDY

- We extended the evidence on integrating data quality interventions within existing systems to improve the quality of facility-based data for monitoring and planning.
- We demonstrated the value of an integrated district-focused data quality intervention to include regular self-assessments of data quality, peer review and feedback, work planning for improvement, and ongoing support through social media.
- We assessed the usefulness of the World Health Organization's catalogue of data quality metrics to measure and monitor the quality of routine facility data, as data quality studies primarily review completeness and accuracy of facility reporting.
- Without a concurrent comparison group, our before-and-after analyses cannot eliminate the effects of concurrent events and activities on data quality metrics.

For peer review only

INTRODUCTION

Routine health information systems provide essential data for governments and stakeholders to make decisions for managing performance and optimizing service delivery.[1-3] Routine health information systems, which include facility-based data, have the potential to provide disaggregated statistics important for understanding disparities and inequities in the provision of quality services and related health outcomes.[4 5]

Effective use of routine health data is dependent, in part, on the quality of data.[1 4 6-9] Studies assessing the quality of routine health data have shown persistent challenges in incomplete and untimely reporting, incomplete indicator-level data, inaccurate facility reporting, and imprecise target population estimates for coverage.[10-13] Further, studies have noted considerably poorer data quality at the facility-level than at district-, state-, and national-levels, citing challenges in accurately capturing data as well as in tallying and summarizing service data for monthly reporting.[14-16]

For peer review only - http://bmjopen.bmj.com/site/about/guidelines.xhtml

Improving the quality of routine data is a priority given the potential of routine data to contribute to effective program monitoring and performance management. Efforts to improve the quality of routine data have included data quality checking with feedback as a low-resource and low-cost activity for strengthening data quality, particularly when implemented as a routine activity. Data quality checking with feedback have been shown to improve completeness, timeliness, and accuracy of facility reporting.[4 6 7 10 17 18] Knowledge transfer activities, such as mentoring, training, or workshops, have offered an opportunity to receive additional skills building in data quality checking or data use. [6 8 19-28] Knowledge sharing activities such as data review meetings and dashboards have brought together different health system-levels or peers within a health system level and include understanding the relative performance of teams on service coverage indicators or on dimensions of data quality.[6 8 20 22 29-31] The introduction of technology, either software such as DHIS 2 or a device to enhance data collection such as a tablet, has demonstrated improvements in completeness, timeliness, and error detection. However, these technology-based initiatives often required supervision, monitoring, and feedback to ensure errors were resolved and that data entered were accurate and consistent.[7 27 32 33] Activities which aligned with user priorities and were integrated within existing government systems were perceived to be advantageous as well as more likely to be adopted and adapted.[20]

Within the context of routine health information systems, the World Health Organization (WHO) has characterized routine data quality into four broad dimensions: completeness and timeliness; internal consistency; external consistency; and external comparisons.[34] While the abovementioned data quality interventions have demonstrated increases in completeness and accuracy of facility reporting[4 6 8 35 36], there are few peer-reviewed studies that quantitatively assessed changes in data quality metrics beyond this.[3 9 37]

In this study, our primary objective was to measure the changes in data quality metrics before and after the introduction of an integrated district-focused intervention in northeastern Nigeria for

For peer review only - http://bmjopen.bmj.com/site/about/guidelines.xhtml

routine facility data captured in primary health facilities. A secondary objective was to determine the extent to which expanding data quality metrics beyond completeness and accuracy of facility reporting offered new insight into reviewing data quality.

METHODS

Study design

This was a before-and-after study design for a data quality intervention in all 11 local government areas (LGA, district-equivalent) of Gombe State, northeastern Nigeria. We present results for the state (n=492 facilities), comparing the 21-month period before the intervention, July 2015-March 2017, with the 21-month period after introducing the intervention, April 2017-December 2018.

Study approvals

Study approvals were obtained from the London School of Hygiene & Tropical Medicine (reference 14091) and the Health Research Ethics Committees for Nigeria (reference NHREC/01/01/2007) and Gombe State (reference ADM/S/658/Vol. II/66).

Patient and public involvement

Patients or the public were not involved in the design, conduct, reporting, or dissemination of the research described here.

Study setting

For peer review only - http://bmjopen.bmj.com/site/about/guidelines.xhtml

Gombe State is located in northeastern Nigeria, a region with high maternal and newborn mortality at 1,549 per 100 000 live births and 35 per 1,000 live births, respectively.[38 39] With an estimated population of 2.9 million, Gombe is predominantly rural and 35% of the women have some primary school education.[40 41] Most women access maternity care through public facilities. Seventy-two percent of women reported at least one antenatal care visit during their last pregnancy and 28% gave birth in a health facility.[39 41] In 2018, over 70% of facility deliveries took place in rural primary health facilities.[42]

Under Nigeria's national policy of *Primary Health Care Under One Roof*, the Gombe State Primary Health Care Development Agency oversees the administration and service delivery for primary health facilities across 11 local government areas (LGA); each LGA has 10-11 political wards (114 wards, total).[43 44] LGA monitoring and evaluation officers are responsible for community- and facility-level data collection, validation, and reporting to the state office. LGA maternal, neonatal, and child health coordinators support the supervision and implementation of services for women and children.

During the intervention period, Gombe State had 492 primary health facilities providing antenatal and childbirth services. As in other states in Nigeria, Gombe facility staff generally completed 13 paper-based registers to document the services they provide (Nigeria Health Management Information Systems, version 2013). Every month, a subset of data in these registers were tallied and summarized in a paper-based report and sent to the LGA health office to be entered into District Health Information Software, version 2 (DHIS2).

Data quality intervention

The routine data quality intervention period spanned 21 months, from April 2017 through December 2018. Following a situational analysis and literature review, development of the intervention took

For peer review only - http://bmjopen.bmj.com/site/about/guidelines.xhtml

place in consultation with the Gombe State Primary Health Care Development Agency. The design was predicated on three key issues. Interventions that include data quality assessments and feedback have demonstrated improvement in completeness, timeliness, and consistency.[4 6 8 19 20 22 23 26-28] Monitoring and evaluation officers and program coordinators in the state were expected to engage with data from facilities to support improvement of service delivery; however, their interactions were not optimally structured to ensure efficient and complementary engagement with facility data. Further, the recent publication of the WHO data quality review toolkit in early 2017 provided clarity on assessing the data quality dimensions and metrics for facility data; however, the Gombe district staff required further support and job aids to administer the data quality reviews as recommended in the WHO guidance.[45]

Thus, the intervention emphasized the partnership between the LGA monitoring and evaluation officer and the LGA maternal, newborn, and child health program coordinator to underscore the link between the quality and use of routine data.[8] The intervention was designed to facilitate existing LGA-level supervision responsibilities. It leveraged scheduling of ongoing activities to minimize cost, added job aids based on the WHO data quality review toolkit, and defined performance standards to provide structure to existing data quality checking duties and to target feedback to facilities.

The intervention included the following activities: (i) data quality learning workshops to present data quality self-assessment findings and develop work plans for improvement; (ii) defining data quality performance standards and milestones for completeness, timeliness, and consistency; (iii) introduction of job aids to self-assess data quality according to the WHO data quality metrics; (iv) monthly state- and LGA-level data quality summary reports; (v) intentional practice on providing constructive feedback to peers and low-performing facilities to promote a positive culture of data use; and (vi) ongoing engagement on data quality issues through government-approved communication channels, including the social media application WhatsApp.

For peer review only - http://bmjopen.bmj.com/site/about/guidelines.xhtml

Twenty-six main attendees participated in the workshops and ongoing communication in-between workshops. These included two participants from each of the 11 LGAs, a monitoring and evaluation officer and the maternal, newborn, and child health program coordinator. At the state-level, four officials participated: the director of the Gombe State Planning, Research, and Statistics Department, the state monitoring and evaluation officer, the state health management information systems officer, and the state maternal, newborn, and child health coordinator.

Four data quality learning workshops took place every six to nine months. The two-day workshops included the introduction of job aids and practical sessions to strengthen data quality checking skills, the presentation of the state's and each LGA's self-assessment of data quality, and the development of six-month work plans to improve the quality of facility-based routine data. Using materials designed for post-graduate learning and teaching, there was intentional practice on how to provide constructive feedback to peers and facilities to promote a positive culture of information use.[46]

At each workshop, a major theme emerged during the work planning sessions (Figure 1). For example, at the first workshop, participants were concerned with inconsistencies observed between the paper-based facility registers and the facility's monthly summary reports. Activities enacted from the work planning session were to revitalize dormant strategies previously set up to address program monitoring and evaluation activities: (i) LGA data validation committee meetings, where facilities bring their registers for verification against their submitted monthly facility report. and (ii) a social media WhatsApp group of LGA actors and facilities. The LGA teams posted pictures and comments on these facility interactions on the WhatsApp group for encouragement and accountability.

After the first workshop, the Gombe State monitoring and evaluation officer disseminated monthly state- and LGA-level data quality summary reports. LGAs were assessed according to the WHO data quality metrics and recommendations for improvement were offered. Initially, this activity was

For peer review only - http://bmjopen.bmj.com/site/about/guidelines.xhtml

designed for the external workshop facilitators to compose and disseminate, while building the capacity of the state officer to take on this task over time.

Outcomes

Using the WHO data quality review toolkit for routine facility data, we assessed 9 metrics across the three data quality dimensions of completeness and timeliness; internal consistency; and external consistency.[45] The data sources and analyses for each data quality metric are described in the following section. Supplementary table S1 provides additional information on each data quality metric assessed and the data sources reviewed.

Data analysis and data sources

Three data sources were used to assess the routine data quality metrics, described below: facility-reported data in DHIS2; external facility surveys; and external household surveys.

DHIS2 contained monthly reports for the 492 primary facilities providing antenatal care, childbirth, and postnatal care services. Monthly aggregated DHIS2 data for July 2015-December 2018 were downloaded at one time and included 14 maternal and newborn health-related data elements. These data were used to assess availability of facility reporting; timeliness of facility reporting; completeness of all 14 priority maternal and newborn health data elements, per monthly facility report; completeness of data element; presence of moderate and extreme outliers; consistency of indicator values over time; and consistency between related data elements.

To assess completeness of facility reporting, we compared the proportions of facilities submitting reports before the intervention and during the intervention. To assess timeliness of facility reporting, the numerator for completeness of facility reporting was further limited to facilities that submitted the reports by the given timelines. To assess both completeness of data-related metrics, we compared the proportions of facility reports where a value was present for the relevant data. To

For peer review only - http://bmjopen.bmj.com/site/about/guidelines.xhtml

compare the proportions of the pre-intervention and intervention periods, we used linear mixed models to account for the clustering of facility measurements within facilities and the clustering of facilities within districts. P-values for these metrics were computed from the Wald test within the mixed models. In these models, we also adjusted for potential confounders such as total client volume and time.[4 6]

We calculated the intraclass correlation coefficient (ICC) as a measure of agreement for the WHO metric assessing consistency between related data elements. ICC values range between 0 and 1, where values approaching 1 represented greater agreement between two related data elements. As the intervention worked through district-level staff to improve data quality across 492 primary health facilities, a paired t-test was carried out to compare pre-intervention and intervention periods matching ICCs at the district-level.

In 2016 and 2018, facility-level surveys were conducted in 97 primary facilities across Gombe to assess their readiness to provide maternal and newborn health services. The two surveys represented the approximate midpoints of the pre-intervention and intervention period. The selected facilities were a state-wide random sample drawn from all primary health facilities. Detailed methods are reported elsewhere.[47] The facility survey protocol was similar to a Service Availability and Readiness Assessment and also included data extraction from the facility's paper-based antenatal and postnatal care register and the labor and delivery register (Nigeria health management information system, version 2013).[48] A trained third party data collection team tallied and recorded the register data for each month of the six-month periods immediately prior to the survey: January-June 2016 and February-July 2018.

These data were used to assess the accuracy of facility reporting (also referred to as data accuracy, data verification, or concordance in peer-reviewed literature). We compared the facilities' paper-based registers data with the facilities' monthly reported data in DHIS2. As with the consistency between related data elements metric, we calculated the ICC as a measure of agreement and carried

For peer review only - http://bmjopen.bmj.com/site/about/guidelines.xhtml

out a paired t-test for the pre-intervention and intervention periods matching ICCs at the district level. ICC values approaching 1 represented greater agreement.

In 2016 and 2018, household-level surveys were conducted in the enumeration areas of the abovementioned 97 primary facilities to assess access to and quality of maternal and newborn services.[47] A total of 79 enumeration areas were surveyed since some enumeration areas were served by more than one facility. All households in each enumeration area were surveyed. The household surveys included a mother's module which asked all women who reported a birth in the last year a detailed set of questions about their contact with health services across the continuum of care from pregnancy to postnatal care. Informed consent was obtained at the community leadership-level and at the individual-level for each respondent. All invited participants agreed to be interviewed.

These data were used for external consistency during the pre-intervention and intervention periods. We compared coverage estimates from household surveys to those from the 97 matching facilities in DHIS2. We compared the same recall period for the surveys and the DHIS2. The DHIS2 data are considered consistent if they fall within the confidence intervals of the external household survey estimates. Calculations of point estimates and their 95% confidence intervals were done using the svyset Stata command (StataCorp, College Station, USA) to adjust for clustering. We chose the highest-order clustering level to provide the most conservative confidence interval estimates.[49]

For peer review only

RESULTS

An integrated district-focused data quality intervention was implemented across 11 LGAs overseeing 492 primary health facilities providing maternal and newborn care services. Below, we present the results for 9 data quality metrics.

For peer review only - http://bmjopen.bmj.com/site/about/guidelines.xhtml

Completeness and timeliness

Table 1 summarizes the completeness and timeliness of reporting at the facility- and indicator-levels.

At the facility-level, the availability of monthly facility reports improved from 72% to 82% (p<0.001) and timeliness of submitting the reports increased from 60% to 72% (p<0.001). The proportion of facility-months where all 14 priority maternal and newborn health data elements contained a value within the monthly report increased from 62% to 68% (p<0.001).

At the indicator-level, seven of 14 data elements assessed improved in completeness compared to the pre-intervention period. Indicator-level completeness did not change for contact indicators such as first antenatal care visits, total antenatal care visits, and facility deliveries.

For peer review only

For peer review only - http://bmjopen.bmj.com/site/about/guidelines.xhtml

Table 1. Facility- and indicator-level completeness and timeliness, Gombe State (n=492 facilities)

	Pre-intervention Jul '15-Mar '17 % (95% CI)	Intervention Apr '17- Dec '18 % (95% CI)
**Facility-level**		
Availability of monthly facility reports	72 (69-74)	82 (80-84)
Timeliness of monthly facility reports	60 (57-62)	72 (70-74)
Completeness of all 14 priority maternal and newborn health data elements, per monthly facility report	62 (60-63)	68 (66-70)
**Indicator-level**		
For every 100 facilities that submitted a monthly facility report, the percentage of facilities reporting a value for the following services:		
First antenatal care visits	76 (67-85)	77 (70-84)
Total antenatal care visits	100 (99-100)	100 (100-100)
Facility deliveries	68 (59-77)	67 (60-74)
For every 100 facilities that reported a value for first ANC visit, the percentage of facilities reporting a value for the following services:		
Antenatal care anemia testing	28 (16-39)	36 (24-49)
Antenatal care syphilis testing	42 (23-61)	29 (23-35)*
Iron-folic acid supplementation	80 (75-84)	89 (85-92)
At least one dose administered of intermittent preventive treatment of malaria	45 (34-56)	56 (49-62)
At least one dose administered of tetanus toxoid vaccine	90 (86-93)	89 (86-91)
For every 100 facilities that reported a value for a facility delivery, the percentage of facilities reporting a value for the following services:		
Delivery by skilled birth attendant	43 (25-61)	86 (81-91)
Live birth or still birth	90 (86-95)	96 (94-97)
Baby weighed at birth	89 (83-95)	95 (94-97)
Oral polio vaccine given at birth	79 (70-87)	86 (82-90)
Early postpartum-postnatal care within 3 days of birth	45 (38-53)	55 (46-64)
Bacillus Calmette-Guérin vaccine given during postnatal care period	79 (71-88)	81 (77-86)

Notes:
* During the intervention period, commodities for antenatal care syphilis testing were redistributed and restricted to 57 facilities. For these 57 facilities, completeness of data for antenatal care syphilis testing increased from 48% (95% CI: 28-68) to 77% (95% CI: 69-86).

For peer review only - http://bmjopen.bmj.com/site/about/guidelines.xhtml

Internal consistency: consistency between related data elements

To assess the consistency between related data elements with a predictable relationship, two types of relationships were reviewed (Table 2). The first type of relationship assessed concurrent tallying across different data elements within and across facility registers. For example, (1) normal deliveries + caesarean deliveries + assisted deliveries = live births + still births; and (2) total postpartum visits reported = sum of the postnatal visit categories reported. For Gombe State, the ICC of delivery types (normal, caesarean, assisted) to birth types (live births, still births) improved from 0.83 (95%CI: 0.73-0.94) to 0.95 (0.93-0.97). Similar patterns of improvement were noted for postnatal visit tallying from an ICC of 0.46 (0.36-0.57) to 0.76 (0.66-0.85).

The second type of relationship assessed was a service provision compared to a contact indicator (e.g., the number of antenatal care syphilis testing done compared to antenatal care first visits, the number of babies weighed at birth compared to the number of facility deliveries). During the pre-intervention period, one of the 10 relationships reflected high consistency: iron-folic acid supplementation. During the implementation period, seven of the 10 relationships reflected improved consistency.

Internal consistency: accuracy of facility reporting

Comparing the facilities' registers with their submitted monthly reports, accuracy of facility reporting (data accuracy) had improved for 6 of 7 indicators, reflecting greater agreement during the intervention period (Table 3). For total antenatal care visits, there was considerable variation between districts during the pre-intervention and intervention periods, with an ICC of 0.62 (95% CI: 0.41-0.83) and 0.86 (95% CI 0.72-0.99), respectively.

For peer review only - http://bmjopen.bmj.com/site/about/guidelines.xhtml

Table 2. Internal consistency: consistency between data elements with a predictable relationship (n=492 facilities)

Data elements with a predictable relationship	ICC, Pre-intervention	ICC, Intervention	P-value
Concurrent tallying within and across facility documentation			
Normal + caesarean + assisted deliveries = livebirths + stillbirths	0.83 (0.73-0.94)	0.95 (0.93-0.98)	0.023
Total postnatal care visits = sum (postnatal care visit categories)	0.46 (0.36-0.57)	0.76 (0.66-0.85)	<0.001
Relationship between first antenatal care visits and:			
Antenatal care anemia testing	0.39 (0.18-0.59)	0.51 (0.35-0.68)	0.016
Antenatal care syphilis testing	0.19 (0.09-0.29)	0.49 (0.37-0.60)	0.003
At least one dose of intermittent preventive treatment of malaria	0.33 (0.17-0.48)	0.43 (0.32-0.54)	0.129
At least one dose of tetanus-toxoid vaccine	0.67 (0.53-0.80)	0.73 (0.65-0.82)	0.167
Relationship between total antenatal care visits and:			
Iron-folic acid supplementation	0.92 (0.89-0.95)	0.97 (0.95-0.99)	0.005
Relationship between facility deliveries and:			
Baby weighed at birth	0.82 (0.71-0.93)	0.94 (0.92-0.97)	<0.001
Delivery by skilled birth attendant	0.42 (0.26-0.59)	0.87 (0.80-0.94)	<0.001
Oral polio vaccine given at birth	0.48 (0.32-0.63)	0.72 (0.65-0.79)	0.007
Early postpartum-postnatal care within 3 days of birth	0.09 (0.06-0.13)	0.19 (0.13-0.24)	<0.001
Bacillus Calmette-Guérin vaccine given during postnatal period	0.56 (0.40-0.72)	0.67 (0.57-0.76)	0.147

Notes:
ICC values range from 0 to 1, with values approaching 1 representing greater agreement.

For peer review only - http://bmjopen.bmj.com/site/about/guidelines.xhtml

Table 3. Accuracy of facility reporting: comparison of paper-based facility records and facility monthly reports in DHIS2 (n=97 facilities)

Data element	ICC, Pre-intervention	ICC, Intervention	P-value
First antenatal care visits	0.57 (0.40-0.75)	0.88 (0.83-0.93)	0.004
Total antenatal care visits	0.62 (0.41-0.83)	0.86 (0.72-0.99)	0.050
Antenatal care syphilis testing	0.08 (-0.02-0.18)	0.59 (0.42-0.76)	<0.001
Facility deliveries	0.48 (0.26-0.70)	0.82 (0.69-0.94)	0.019
Use of partograph to monitor labor and delivery	0.24 (0.03-0.45)	0.83 (0.77-0.89)	<0.001
Total postnatal care visits	0.15 (0.02-0.29)	0.58 (0.38-0.78)	0.001
Early postpartum-postnatal care within 3 days of birth	0.21 (0.01-0.41)	0.55 (0.31-0.80)	0.020

Notes:
ICC values range from 0 to 1, with values approaching 1 representing greater agreement.

Internal consistency: outliers and consistency over time

Supplementary tables S2 and S3 summarize the presence of outliers and the consistency over time for the 14 maternal and newborn data elements. The frequency of months when outliers were reported decreased during the intervention period. However, 11 moderate outliers were reported during the intervention period compared to eight moderate outliers and two extreme outliers reported during the pre-intervention period. All 11 outliers reported during the intervention period occurred in May 2018 during a health worker strike. Of the 14 data elements assessed, six data elements were inconsistent over time due to reported increases in services when comparing the final year of the intervention 2018 to the mean value of the last 3 years and when comparing the pre-intervention and intervention periods.

For peer review only - http://bmjopen.bmj.com/site/about/guidelines.xhtml

External consistency: agreement between facility summary reports in DHIS2 and household surveys

Figure 2 summarizes external consistency, which is the agreement between facility-based routine data in DHIS2 compared to household-level surveys in the enumeration areas of these facilities. Other than the indicator for early postpartum-postnatal care, there was no agreement nor any consistent pattern of agreement between facility-based routine data and the household surveys. DHIS2 data underestimated compared to the household survey for at least one dose of intermittent preventative therapy for malaria in pregnancy and at least one dose of tetanus toxoid. DHIS2 data overestimated compared to the household survey for baby weighed at birth, oral polio vaccine given at birth, and Bacillus Calmette-Guérin given during postnatal period. For antenatal care anemia testing, facility-based estimates were within the household survey estimate confidence interval but overestimated compared to the household survey during the intervention period.

DISCUSSION

Facility-based routine data are an important source for monitoring, performance management, and planning.[9] Our study found that an integrated district-focused data quality intervention – which included regular self-assessment of data quality, peer review and feedback, learning workshops, work planning for improvement, monthly data quality reports, and ongoing support through social media – was associated with improvements across most WHO data quality metrics. There were differences in data quality improvement by data element type.[50] Data related to content of care or the provision of commodities, such as syphilis testing and intermittent preventative therapy for malaria, improved more across data quality metrics compared to contact indicators. Contact indicators had relatively higher data quality metrics before the intervention, such as first antenatal

For peer review only - http://bmjopen.bmj.com/site/about/guidelines.xhtml

care visits and facility deliveries. Contact indicators such as first antenatal care visits, fourth antenatal care visits, and facility deliveries are generally well-defined events to document, have been key denominators for local program planning, and have been prioritized for monitoring progress in previous global initiatives including the Millennium Development Goals and Countdown to 2015.[50-52]

This was an integrated data quality intervention designed to facilitate existing state- and district-level data quality checking responsibilities and emphasize the partnership between the monitoring and evaluation officers and the maternal, newborn and child health program coordinators to expand local access to the DHIS2 data, use the data, and problem solve.[9] Incorporating regular data quality reviews and feedback within supervision is an acknowledged low-cost and effective activity for improving completeness and consistency.[6 8 17 18 53] Leveraging existing staff and meeting schedules as well as adding structure to existing data-related responsibilities further promotes the feasibility of this intervention in similar settings. The evolution of the intervention through the work planning sessions prompted local solutions defined by the participants as feasible and within their resources to implement. In particular, the participants' decision to revitalize the data validation committees during the first workshop engaged the facilities early on to ensure the facility's register counts matched the facility's monthly report aggregate value. This early engagement with facilities could have contributed to the increased ICCs for accuracy of facility reporting observed during the intervention period (as shown in Table 3). A formative phase of the intervention might have captured activities such as the data validation committee as a pre-defined intervention activity. Including a formative phase should be a consideration for future implementation.

Our findings aligned with previous studies reporting improvements in completeness, timeliness, and accuracy of facility reporting after intervention. However, this study's relative gains may reflect the scale of working through 11 districts with 492 primary health facilities.[4 6 8 35 36] A data quality intervention in KwaZulu-Natal province, South Africa, which included trainings, monthly data

For peer review only - http://bmjopen.bmj.com/site/about/guidelines.xhtml

meetings, and external data quality audits across 78 facilities improved completeness of six data elements from 26% to 64% and the agreement between facility records and reports (data accuracy) from 37% to 65%.[6] A province-wide data quality intervention in Sofala, Mozambique, for 26 facilities included regular district-level review meetings for health workers and managers, data dashboards for tracking trends and rankings, human resource optimization models, and equipment purchase and maintenance. The summary measure used to evaluate data quality improvement, concordance, improved from 56% to 88% during the intervention period.[4] The introduction of an electronic medical record to support data quality improvement in 27 facilities across Kenya recorded a decline in missing data from 31% to 13% for 24 data elements, with a mean concordance score increasing across facilities by 1.79 (95% CI:0.25-3.33).[36]

While our findings align with previous studies for increased completeness, timeliness, and accuracy, our study reviewed additional WHO metrics to give a more comprehensive picture of the dimensions of data quality. This study also provided an opportunity to reflect on the relative usefulness of assessing all WHO data quality metrics to understand the quality of routine data in a given context. Sharp increases in service uptake due to health campaigns or targeted health projects make the assessment of moderate outliers and consistency over time less insightful about data quality, especially in the context of urgent efforts towards achieving universal health coverage. Our study found that, other than early postpartum-postnatal care within three days of birth, there were no instances of agreement between the facility-based routine data and external household surveys. However, an emerging body of criterion validity studies have demonstrated mixed results in the ability of women to recall facility-based pregnancy- and childbirth-related events in household surveys.[54-58] More research is needed on how to reconcile health facility and household survey data, while also reconsidering the emphasis on the household surveys as the reference standard.

The data quality metric to assess the consistency between related data elements provided useful insights in addition to the completeness and accuracy regularly reported in the literature. Assessing

For peer review only - http://bmjopen.bmj.com/site/about/guidelines.xhtml

the data relationship for a service provision compared to a contact indicator (e.g., ANC anemia testing compared to ANC first visits) allows for discussion on whether observed discrepancies are due to low service uptake or poor reporting, an important consideration given the emphasis on improving quality of care and understanding effective coverage.[59] Further, assessing data relationships that require concurrent tallying of services/information across data sources (e.g. facility attendance = inpatient + outpatient; normal delivery + caesarean delivery + assisted delivery = live births + still births), provides useful insight about whether a facility is paying attention to the internal consistency of their data within and across facility documentation. Focus on accuracy of facility reporting, a more common metric assessed in the peer-reviewed literature (referred to as data accuracy or concordance), is an important data quality metric as subnational-, national-, and global-level monitoring cannot take place effectively without the confidence that the facilities have summarized and tallied the data as intended. However, this focus on the accuracy of facility reporting up through the different levels of the health system do not require that these data be internally consistent with other data.

Our study had limitations. Without a concurrent comparison group, our before-and-after analyses cannot eliminate the effects of concurrent events and activities on data quality metrics. It is possible that other activities contributed to the observed data quality improvements. Given the high burden of maternal and neonatal mortality, the Gombe State Primary Health Care Development Agency spearheaded an initiative to improve maternal and neonatal services with the aim of having one fully functional primary health facility in each of its 114 wards. During the intervention period, 57 facilities (12% of the 492 maternity facilities) received support including facility-level quality improvement support as well as community-based outreach and education to increase uptake of services. Facility-level activities included support on data quality to monitor trends in services provided and the provision of computers and facility registers. Additionally, similar to other data quality assessments, we did not validate the data through direct clinical observations[4 6 10 12 32 60 61] nor did we compare the paper-based monthly summary reports to their electronic versions in DHIS2[6 12 37 62-64]. Despite close

For peer review only - http://bmjopen.bmj.com/site/about/guidelines.xhtml

attention to quality control, the facility- and household-level surveys might still be susceptible to errors in data recording, including incorrectly tallying the number of events in the original facility registers for comparison with data in DHIS2.

Improving the quality of routine facility data is essential for local and national evidence-based monitoring of universal health coverage. We found that an integrated district-focused data quality intervention was associated with increases across most WHO data quality metrics for routine facility-based data. Future initiatives should aim to incorporate national- and higher subnational-levels of the health system to determine scalability and sustainability of integrated data quality interventions in the long-term.

Acknowledgement

The authors wish to acknowledge the leadership of the Gombe State Primary Health Care Development Agency throughout the development and implementation of the data quality intervention. We also appreciate the cooperation of and partnership with the LGA M&E officers, MNCH coordinators, and Gombe State implementing partners to improve the quality of routine maternal and newborn health data in Gombe State. We are grateful to the women who participated in the household surveys and to the team who conducted the facility and household data collection. Finally, we are grateful to the facility teams responsible for providing and documenting their care for women and newborns.

Contributors

AAB and TM conceived and designed the study. AAB and EA designed the analyses. AAB carried out the analyses and composed the initial draft. JS, TM, EA, NU, AA, HF reviewed the early drafts. All authors approved the final draft.

For peer review only - http://bmjopen.bmj.com/site/about/guidelines.xhtml

Data sharing statement

Data for this study are available from the London School of Hygiene & Tropical Medicine public repository: http://datacompass.lshtm.ac.uk/229/.

Funding

This work was supported by the Bill & Melinda Gates Foundation [OPP1149259]. The funder of this study had no role in the study's design or conduct, data collection, analysis or interpretation of results, writing of the paper, or decision to submit for publication.

Competing interests

Mr Felix Habila and Mr Ahmed Audu are members of the Gombe State Primary Health Care Development Agency. The authors declare no other competing interests.

For peer review only

For peer review only - http://bmjopen.bmj.com/site/about/guidelines.xhtml

References

1. Nutley T. Improving data use in decision making: an intervention to strengthen health systems. Chapel Hill, North Carolina: MEASURE Evaluation, 2012.
2. AbouZahr C, Boerma T. Health information systems: the foundations of public health. *Bull World Health Organ* 2005;83(8):578-83.
3. Nisingizwe MP, Iyer HS, Gashayija M, et al. Toward utilization of data for program management and evaluation: quality assessment of five years of health management information system data in Rwanda. *Global Health Action* 2014;7:25829. doi: http://dx.doi.org/10.3402/gha.v7.25829
4. Wagenaar BH, Gimbel S, Hoek R, et al. Effects of a health information system data quality intervention on concordance in Mozambique: time-series analyses from 2009–2012. *Population Health Metrics* 2015;13(1):9. doi: 10.1186/s12963-015-0043-3
5. Chilundo B, Sundby J, Aanestad M. Analysing the quality of routine malaria data in Mozambique. *Malar J* 2004;3(3) doi: 10.1186/1475-2875-3-3
6. Mphatswe W, Mate KS, Bennett B, et al. Improving public health information: a data quality intervention in KwaZulu-Natal, South Africa. *Bull World Health Organ* 2012;90(3):176-82. doi: https://dx.doi.org/10.2471/BLT.11.092759
7. Mutale W, Chintu N, Amoroso C, et al. Improving health information systems for decision making across five sub-Saharan African countries: implementation strategies from the African Health Initiative. *BMC Health Serv Res* 2013;13(2):S9. doi: 10.1186/1472-6963-13-S2-S9
8. Braa J, Heywood A, Sahay S. Improving quality and use of data through data-use workshops: Zanzibar, United Republic of Tanzania. *Bull World Health Organ* 2012;90(5):379-84. doi: https://dx.doi.org/10.2471/BLT.11.099580
9. Maïga A, Jiwani SS, Mutua MK, et al. Generating statistics from health facility data: the state of routine health information systems in Eastern and Southern Africa. *BMJ Global Health* 2019;4(5):e001849. doi: 10.1136/bmjgh-2019-001849
10. Bosch-Capblanch X, Ronveaux O, Doyle V, et al. Accuracy and quality of immunization information systems in forty-one low income countries. *Trop Med Int Health* 2009;14(1):2-10. doi: 10.1111/j.1365-3156.2008.02181.x
11. Ronveaux O, Rickert D, Hadler S, et al. The immunization data quality audit: verifying the quality and consistency of immunization monitoring systems. *Bull World Health Organ* 2005;83(7):503-10.
12. Mate KS, Bennett B, Mphatswe W, et al. Challenges for routine health system data management in a large public program to prevent mother-to-child HIV transmission in South Africa. *PLoS One* 2009;4(5):e5483.
13. Maina I, Wanjala P, Soti D, et al. Using health-facility data to assess subnational coverage of maternal and child health indicators, Kenya. *Bull World Health Organ* 2017;95(10):683-94. doi: 10.2471/BLT.17.194399
14. Belay H, Lippeveld T. Inventory of PRISM framework and tools: application of PRISM tools and interventions for strengthening routine health information system performance. Working Paper Series No: WP-13-138. Chapel Hill, North Carolina, USA: MEASURE Evaluation, 2013.
15. Hotchkiss DR, Aqil A, Lippeveld T, et al. Evaluation of the Performance of Routine Information System Management (PRISM) framework: evidence from Uganda. *BMC Health Serv Res* 2010;10:188.
16. Aqil A, Avila JE, Mejia LSP, et al. Guanajuato SINAIS assessment. Technical Report No: SR-10-60. Chapel Hill, North Carolina, USA: MEASURE Evaluation, 2010.
17. Lippeveld T, Sauerborn R, Bodart C, et al. Design and implementation of health information systems. Geneva: World Health Organization 2000.

For peer review only - http://bmjopen.bmj.com/site/about/guidelines.xhtml

18. Ledikwe JH, Grignon J, Lebelonyane R, et al. Improving the quality of health information: a qualitative assessment of data management and reporting systems in Botswana. *Health Res Policy Syst* 2014;12:7.
19. Admon AJ, Bazile J, Makungwa H, et al. Assessing and improving data quality from community health workers: a successful intervention in Neno, Malawi. *Public Health Action* 2013;3 doi: 10.5588/pha.12.0071
20. Gimbel S, Mwanza M, Nisingizwe MP, et al. Improving data quality across 3 sub-Saharan African countries using the Consolidated Framework for Implementation Research: results from the African Health Initiative. *BMC Health Serv Res* 2017;17(Suppl 3):828. doi: 10.1186/s12913-017-2660-y
21. Karemere JN. Centers of excellence in monitoring and evaluation: an approach to improving data quality for effective decision making in the Democratic Republic of the Congo. *Am J Trop Med Hyg* 2017;97(5 Supplement 1):284-85.
22. Orji B, Olisaekee G, Udenze O, et al. Improving quality of data to advance malaria in pregnancy indicator coverage in Ebonyi State, Nigeria. *Am J Trop Med Hyg* 2017;97(5 Supplement 1):335.
23. Westercamp N, Staedke S, Hutchinson E, et al. Effectiveness and sustainability of a collaborative improvement method to increase the quality of routine malaria surveillance data in Kayunga District, Uganda. *Am J Trop Med Hyg* 2017;97(5 Supplement 1):31.
24. Kamadjeu RM, Tapang EM, Moluh RN. Designing and implementing an electronic health record system in primary care practice in sub-Saharan Africa: a case study from Cameroon. *Inform Prim Care* 2005;13(3):179-86.
25. Barrington J, Wereko-Brobby O, Ward P, et al. SMS for Life: a pilot project to improve anti-malarial drug supply management in rural Tanzania using standard technology. *Malar J* 2010;9 doi: 10.1186/1475-2875-9-298
26. Ledikwe JH, Reason LL, Burnett SM, et al. Establishing a health information workforce: innovation for low- and middle-income countries. *Human Resources for Health* 2013;11:35. doi: https://dx.doi.org/10.1186/1478-4491-11-35
27. Kiberu VM, Matovu JKB, Makumbi F, et al. Strengthening district-based health reporting through the district health management information software system: the Ugandan experience. *BMC Med Inform Decis Mak* 2014;14(1):40. doi: 10.1186/1472-6947-14-40
28. Ye M, N'Gbichi JM, Franchard T, et al. Malaria data quality and use in selected centers of excellence in Madagascar: results from a cross-sectional baseline survey. *Am J Trop Med Hyg* 2019;101(5 Supplement):118-19. doi: http://dx.doi.org/10.4269/ajtmh.abstract2019
29. Sherr K, Cuembelo F, Michel C, et al. Strengthening integrated primary health care in Sofala, Mozambique. *BMC Health Serv Res* 2013;13 Suppl 2:S4. doi: https://dx.doi.org/10.1186/1472-6963-13-S2-S4
30. Dodo M, Badolo O, Nebie S, et al. Contribution of quarterly malaria data review and validation to data quality and malaria services improvement. *Am J Trop Med Hyg* 2018;99(4 Supplement):118.
31. Tuti T, Bitok M, Malla L, et al. Improving documentation of clinical care within a clinical information network: an essential initial step in efforts to understand and improve care in Kenyan hospitals. *BMJ Global Health* 2016;1(1):e000028. doi: 10.1136/bmjgh-2016-000028
32. Puttkammer N, Baseman JG, Devine EB, et al. An assessment of data quality in a multi-site electronic medical record system in Haiti. *Int J Med Inform* 2016;86:104-16.
33. Maokola W, Willey BA, Shirima K, et al. Enhancing the routine health information system in rural southern Tanzania: successes, challenges and lessons learned. *Trop Med Int Health* 2011;16(6):721-30.
34. MEASURE Evaluation. Routine health information systems: a curriculum on basic concepts and practices. Chapel Hill, North Carolina, USA: MEASURE Evaluation, 2017.

For peer review only - http://bmjopen.bmj.com/site/about/guidelines.xhtml

35. Gething P, Noor A, Gikandi P, et al. Improving imperfect data from health management information systems in Africa using space-time geostatistics. *PLoS Med* 2006;3 doi: 10.1371/journal.pmed.0030271
36. Muthee V, Bochner AF, Osterman A, et al. The impact of routine data quality assessments on electronic medical record data quality in Kenya. *PLoS One* 2018;13(4):e0195362. doi: https://dx.doi.org/10.1371/journal.pone.0195362
37. Gimbel S, Micek M, Lambdin B, et al. An assessment of routine primary care health information system data quality in Sofala Province, Mozambique. *Population Health Metrics* 2011;9:12.
38. Izugbara CO, Wekesah FM, Adedini SA. Maternal health in Nigeria: a situation update. Nairobi, Kenya: African Population and Health Research Center, 2016.
39. National Bureau of Statistics and United Nations Children’s Fund. Multiple Indicator Cluster Survey 2016-17: Survey Findings Report. Abuja, Nigeria: National Bureau of Statistics and United Nations Children’s Fund, 2017.
40. Gombe State Government. History of Gombe State, Nigeria 2018 [Available from: http://gombestate.gov.ng/history-2/ accessed September 9, 2019.
41. National Population Commission [Nigeria] and ICF International. Nigeria Demographic and Health Survey 2018, Final Report. Abuja, Nigeria, and Rockville, Maryland, USA: NPC/Nigeria and ICF International, 2019.
42. Nigeria District Health Information System version 2. Nigeria Health Management Information System, version 2013: Gombe State facility deliveries, by facility type, January-December 2018. Abuja: Nigeria District Health Information System, version 2, 2019.
43. Federal Government of Nigeria. Integrating primary healthcare governance in Nigeria: implementation manual, 2013.
44. National Primary Health Care Development Agency. Integrating primary health care governance in Nigeria (Primary Health Care Under One Roof). Abuja: NPHCDA, 2013.
45. World Health Organization. Data quality review: a toolkit for facility data quality assessment. Geneva: World Health Organization, 2017.
46. Lippeveld T. Routine health facility and community information systems: creating an information use culture. *Global Health: Science and Practice* 2017;5(3):338–40.
47. Marchant T. Change in maternal and newborn health care. Interactions between families and frontline workers – their frequency, quality, and equity – and coverage of interventions for mothers and newborns. Report from Gombe State, Nigeria, 2012-2015. IDEAS Project Report: London School of Hygiene and Tropical Medicine, 2016.
48. World Health Organization. Service Availability and Readiness Assessment (SARA): an annual monitoring system for service delivery. Reference Manual, Version 2.2. Geneva: World Health Organization, 2015.
49. Bottomley C, Kirby MJ, Lindsay SW, et al. Can the buck always be passed to the highest level of clustering? *BMC Med Res Methodol* 2016;16:29-29. doi: 10.1186/s12874-016-0127-1
50. Bhattacharya AA, Umar N, Audu A, et al. Quality of routine facility data for monitoring priority maternal and newborn indicators in DHIS2: a case study from Gombe State, Nigeria. *PLoS One* 2019;14(1):e0211265. doi: https://dx.doi.org/10.1371/journal.pone.0211265
51. Bryce J, Requejo J, Countdown to 2015 Working Group. Countdown to 2015: tracking progress in maternal, newborn, and child survival. The 2008 report. New York, USA: UNICEF, 2008.
52. World Health Organization. Countdown to 2015: a decade of tracking progress for maternal, newborn and child survival, final report. Geneva: World Health Organization, 2015.
53. Wagenaar BH, Hirschhorn LR, Henley C, et al. Data-driven quality improvement in low-and middle-income country health systems: lessons from seven years of implementation experience across Mozambique, Rwanda, and Zambia. *BMC Health Serv Res* 2017;17 doi: 10.1186/s12913-017-2661-x
54. Stanton CK, Rawlins B, Drake M, et al. Measuring coverage in MNCH: testing the validity of women's self-report of key maternal and newborn health interventions during the

For peer review only - http://bmjopen.bmj.com/site/about/guidelines.xhtml

peripartum period in Mozambique. *PLoS One* 2013;8(5):e60694. doi: 10.1371/journal.pone.0060694
55. Blanc AK, Warren C, McCarthy KJ, et al. Assessing the validity of indicators of the quality of maternal and newborn health care in Kenya. *Journal of Global Health* 2016;6(1):010405. doi: 10.7189/jogh.06.010405
56. Blanc AK, Diaz C, McCarthy KJ, et al. Measuring progress in maternal and newborn health care in Mexico: validating indicators of health system contact and quality of care. *BMC Pregnancy Childbirth* 2016;16(1):255. doi: 10.1186/s12884-016-1047-0
57. McCarthy KJ, Blanc AK, Warren CE, et al. Women's recall of maternal and newborn interventions received in the postnatal period: a validity study in Kenya and Swaziland. *Journal of Global Health* 2018;8(1):010605. doi: 10.7189/jogh.08.010605 [published Online First: 2018/05/12]
58. Bhattacharya AA, Allen E, Umar N, et al. Monitoring childbirth care in primary health facilities: a validity study in Gombe State, northeastern Nigeria. *Journal of Global Health* 2019;9(2):020411. doi: 10.7189/jogh.09.020411
59. Amouzou A, Leslie HH, Ram M, et al. Advances in the measurement of coverage for RMNCH and nutrition: from contact to effective coverage. *BMJ Global Health* 2019;4(Suppl 4):e001297. doi: 10.1136/bmjgh-2018-001297
60. Glèlè Ahanhanzo Y, Ouendo E-M, Kpozehouen A, et al. Data quality assessment in the routine health information system: an application of the lot quality assurance sampling in Benin. *Health Policy Plan* 2015;30(7):837-43.
61. Makombe SD, Hochgesang M, Jahn A, et al. Assessing the quality of data aggregated by antiretroviral treatment clinics in Malawi. *Bull World Health Organ* 2008;86(4):310-4.
62. Nicol E, Dudley L, Bradshaw D. Assessing the quality of routine data for the prevention of mother-to-child transmission of HIV: an analytical observational study in two health districts with high HIV prevalence in South Africa. *Int J Med Inform* 2016;95:60-70.
63. Mlambo MG, Peltzer K, Koivu A. Prevention of mother-to-child transmission of HIV data completeness and accuracy assessment in health facilities of the Nkangala District. *Health SA Gesondheid* 2014;19(1):a774.
64. Omoleke SA, Tadesse MG. A pilot study of routine immunization data quality in Bunza Local Government area: causes and possible remedies. *The Pan African Medical Journal* 2017;27:239. doi: https://dx.doi.org/10.11604/pamj.2017.27.239.11875

For peer review only

For peer review only - http://bmjopen.bmj.com/site/about/guidelines.xhtml

Figures

Figure 1. Data quality learning workshops in Gombe State, April 2017-December 2018

Figure 2. External consistency: comparison of household-level survey and facility summary reports in DHIS2 (n=97 facilities)

For peer review only

For peer review only - http://bmjopen.bmj.com/site/about/guidelines.xhtml

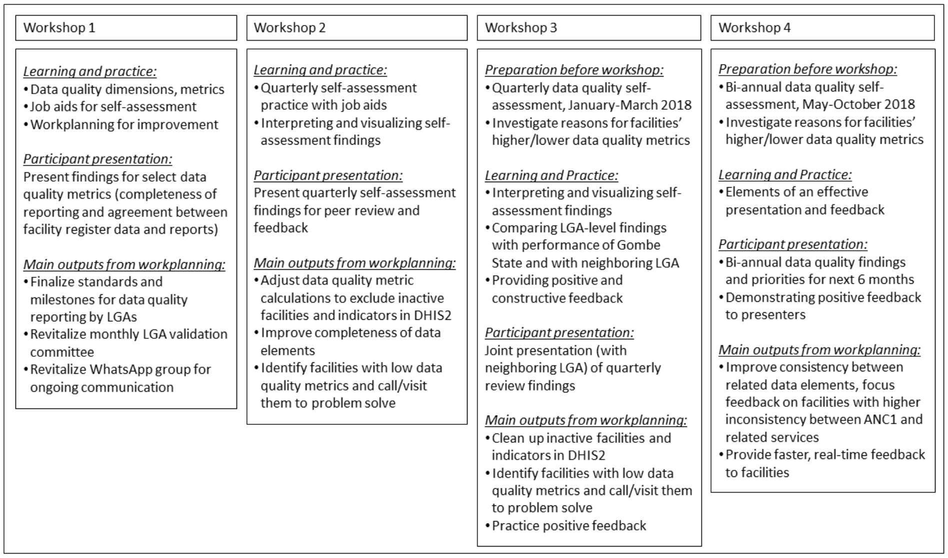

Figure 1. Data quality learning workshops in Gombe State, April 2017-December 2018

54x30mm (600 x 600 DPI)

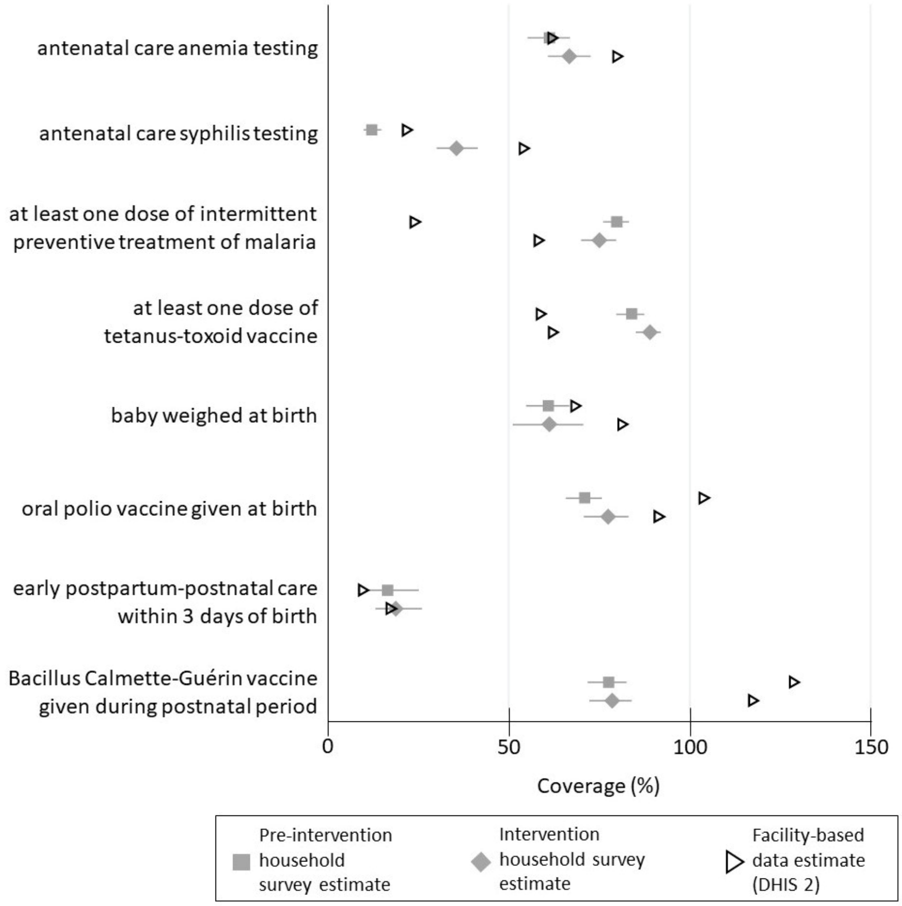

Figure 2. External consistency: comparison of household-level survey and facility summary reports in DHIS2 (n=97 facilities)

87x80mm (300 x 300 DPI)

Supplementary data

Table S1. Data quality metrics and data sources reviewed

Data quality metric	Analysis/calculation	Source(s)
**Data quality dimension 1: Completeness and timeliness**		
Facility-level metrics		
Availability of monthly facility reports	Proportion of facility's expected monthly reports actually submitted	facility monthly reports (DHIS2)
Timeliness of monthly facility reports	Proportion of facility's expected monthly reports actually submitted on time	facility monthly reports (DHIS2)
Completeness of all 14 priority maternal and newborn health data elements, per monthly facility report	Proportion of facility's submitted reports that have a value reported for all 14 priority maternal and newborn health data elements	facility monthly reports (DHIS2)
Indicator-level metrics		
Completeness of data element	Proportion of non-missing values for a given data element in expected monthly reports	facility monthly reports (DHIS2)
**Data quality dimension 2: Internal consistency**		
Consistency over time	Ratio of value of indicator for the intervention period to the mean of preceding 3 years	facility monthly reports (DHIS2)
Outliers	Number and frequency of moderate outliers (±2-3SD from the mean) and extreme outliers (±3SD from the mean) of monthly values	facility monthly reports (DHIS2)
Consistency between related data elements	Reliability index (intraclass correlation coefficient, ICC) comparing two sets of related data elements	facility monthly reports (DHIS2)
Accuracy of facility reporting (data accuracy, concordance)	Reliability index (intraclass correlation coefficient, ICC) comparing original facility register count to monthly facility report in DHIS2	facility registers; facility monthly reports (DHIS2)
**Data quality dimension 3: External consistency**		
Consistency between household surveys and monthly facility reports	Ratio of indicator values in household surveys for facility catchment areas to matching facilities in DHIS2	household surveys; facility monthly reports (DHIS2)

Notes:
DHIS2=District Health Information Software version 2, SD=standard deviation, ICC=intraclass correlation coefficient.

For peer review only

S2. Consistency over time, 2015-2018 (Gombe State, n=492 facilities)

Indicator, data element		2015	2016	2017	2018	Mean (2015-2017)	Ratio of 2018 to Mean (2015-2017)	Mean (2015-2016)	Ratio of Mean (2017-2018) to Mean (2015-2016)
Main denominators									
First antenatal care visits		108,136	131,263	132,715	128,924	124,038	1.04	119,700	1.09
Total antenatal care visits		194,141	365,251	351,596	300,404	303,663	0.99	279,696	1.17
Facility deliveries		59,731	48,335	65,211	52,122	57,759	0.90	54,033	1.09
Data elements									
Antenatal care anemia testing	Number	47,809	58,514	68,189	97,128	58,171	**1.67**	53,162	**1.55**
	Coverage	44%	45%	51%	75%	47%	**1.61**	44%	**1.43**
Antenatal care syphilis testing	Number	26,231	24,569	34,461	47,393	28,420	**1.67**	25,400	**1.61**
	Coverage	24%	19%	26%	37%	23%	**1.60**	21%	**1.46**
Iron-folic acid supplementation	Number	172,447	317,420	248,404	282,221	246,090	1.15	244,934	1.08
	Coverage	89%	87%	71%	94%	82%	1.14	88%	0.94
At least one dose of intermittent preventive treatment of malaria	Number	32,960	27,209	30,292	76,482	30,154	**2.54**	30,085	**1.77**
	Coverage	30%	21%	23%	59%	25%	**2.40**	26%	**1.60**
At least one dose of tetanus toxoid vaccine	Number	69,374	76,754	83,058	85,293	76,395	1.12	73,064	1.15
	Coverage	64%	58%	63%	66%	62%	1.07	61%	1.05
Delivery by skilled birth attendant	Number	18,912	10,240	32,725	47,042	20,626	**2.28**	14,576	**2.74**
	Coverage	32%	21%	50%	90%	34%	**2.63**	26%	**2.66**
Live births and still births	Number	33,492	39,892	47,715	49,053	40,366	1.22	36,692	1.32
	Coverage	56%	83%	73%	94%	70%	1.35	69%	1.21
Baby weighed at birth	Number	32,719	39,145	46,850	48,162	39,571	1.22	35,932	1.32
	Coverage	55%	81%	72%	92%	69%	**1.34**	68%	**1.21**
Oral polio vaccine at birth	Number	50,587	60,636	66,895	64,457	59,373	1.09	55,612	1.18
	Coverage	85%	125%	103%	124%	104%	1.19	105%	1.08
Early postpartum-postnatal care within 3 days of birth	Number	3,930	6,265	8,575	12,868	6,257	**2.06**	5,098	**2.10**
	Coverage	7%	13%	13%	25%	11%	**2.27**	10%	**1.94**
Bacillus Calmette-Guérin vaccine given during postnatal period	Number	75,491	84,670	87,525	81,362	82,562	0.99	80,081	1.05
	Coverage	126%	175%	134%	156%	145%	1.07	151%	0.96

For peer review only

S3. Presence of outliers in facility summary reports in DHIS2 in Gombe State, July 2015-December 2018 (n=492 facilities)

	July 2015-March 2017		April 2017-Dec 2018	
**Indicator, data element**	**Moderate Outliers**	**Extreme Outliers**	**Moderate Outliers**	**Extreme Outliers**
First antenatal visits	1 (Jan 2017)	0	1 (May 2018)	0
Total antenatal visits	1 (Oct 2016)	0	1 (May 2018)	0
Facility deliveries	0	0	1 (May 2018)	0
Antenatal care anemia testing	0	0	1 (May 2018)	0
Antenatal care syphilis testing	0	0	0	0
Iron-folic acid supplementation	0	1 (Oct 2016)	1 (May 2018)	0
At least one dose of intermittent preventive treatment of malaria	0	1 (Oct 2016)	0	0
At least one dose of tetanus toxoid vaccine	1 (Oct 2016)	0	1 (May 2018)	0
Delivery by skilled birth attendant	1 (Mar 2017)	0	1 (May 2018)	0
Live births and still births	1 (Mar 2017)	0	1 (May 2018)	0
Baby weighed at birth	1 (Mar 2017)	0	1 (May 2018)	0
Oral polio vaccine at birth	0	0	1 (May 2018)	0
Early postpartum-postnatal care within 3 days of birth	1 (Jan 2017)	0	0	0
Bacillus Calmette-Guérin vaccine given during postnatal period	1 (Jan 2017)	0	1 (May 2018)	0
**TOTAL OUTLIERS**	**8**<br>2 (Oct 2016),<br>3 (Jan 2017),<br>3 (Mar 2017)	**2**<br>2 (Oct 2016)	**11**<br>11 (May 2018)	**0**<br>0
